# Supplementary material for: Phosphorylation of Histone H4T80 Triggers DNA Damage Checkpoint Recovery
Source: Mol Cell. 2018 Nov 15;72(4):625–635.e4. doi: 10.1016/j.molcel.2018.09.023 (PMC6242705; doi:10.1016/j.molcel.2018.09.023)
Supplement: Document S1. Figures S1–S7 and Table S1 [file mmc1.pdf]

**Molecular Cell, Volume *72***

# Supplemental Information

# Phosphorylation of Histone H4T80

# Triggers DNA Damage Checkpoint Recovery

**Gonzalo Millan-Zambrano, Helena Santos-Rosa, Fabio Puddu, Samuel C. Robson, Stephen P. Jackson, and Tony Kouzarides**

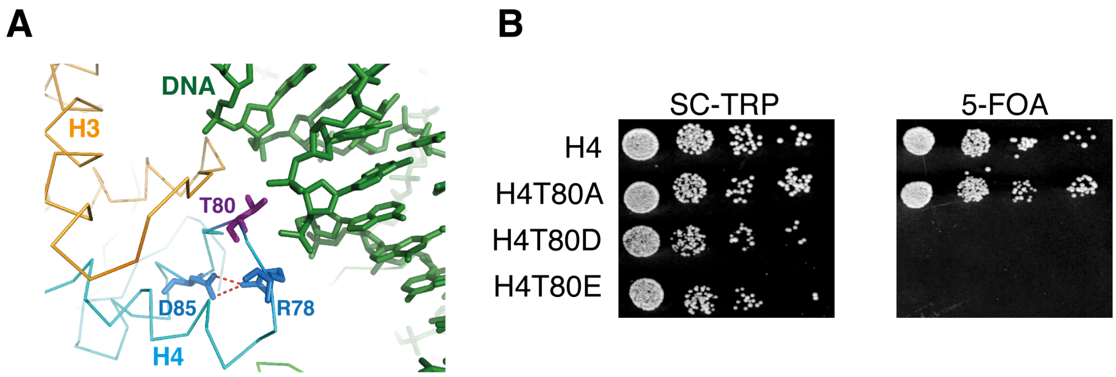


**Figure S1. Histone H4T80 phosphorylation levels must be tightly regulated. Related to Figure 1. (A)** Ribbon structure of H3 L1 loop and H4 L2 loop indicating hydrogen bonds between H4R78 and H4D85. **(B)** Spot test for cell viability. 10-fold serial dilutions were used.

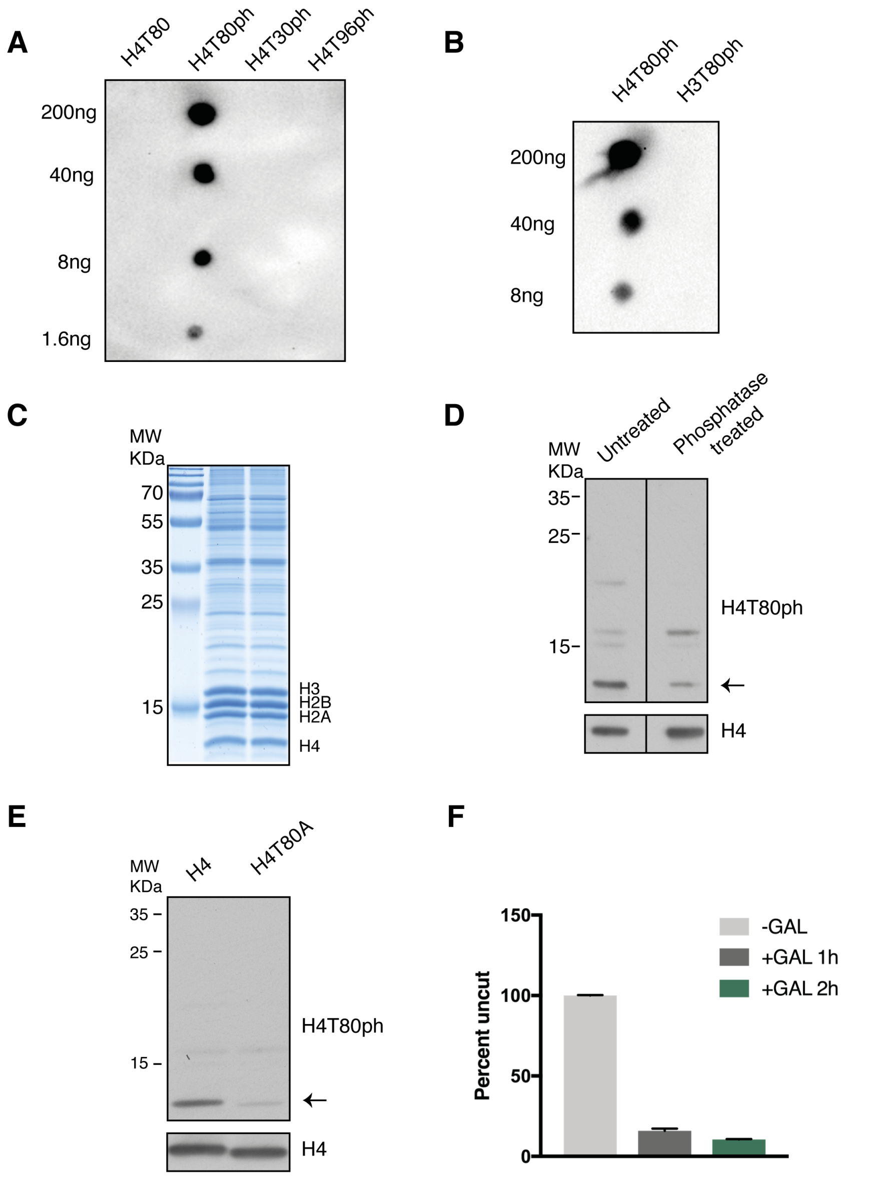


**Figure S2. Anti-H4T80ph antibody specifically recognizes H4T80ph in yeast. Related to Figure 1. (A)** and **(B)** Dot-blot analysis using H4T80ph specific antibody. 5-fold serial dilutions of different peptides, as indicated, were spotted onto a PVDF membrane. **(C)** Coomassie blue stained gel showing purified yeast histones separated by SDS-PAGE in 17% acrylamide gels. **(D)** Immunoblot analysis of purified yeast histones separated by SDS-PAGE in 17% acrylamide gels. Nitrocellulose membranes were treated or not with alkaline phosphatase. Blots were probed with anti-H4T80ph antibody and then re-probed with anti-H4 antibody as indicated. ← Corresponds to histone H4. **(E)** Immunoblot analysis of purified yeast histones separated by SDS-PAGE in 17% acrylamide gels. Both wild type and H4T80A mutant cells were grown to exponential phase. Blots were probed with anti-H4T80ph antibody and then re-probed with anti-H4 antibody as indicated. **(F)** HO cutting efficiency was measured by qPCR using primers encompassing the HO cut site and normalized to the *PRP8* reference locus. The mean of the non-induced samples was calculated as 100% uncut. Graphs show mean +SEM of three biological replicates.

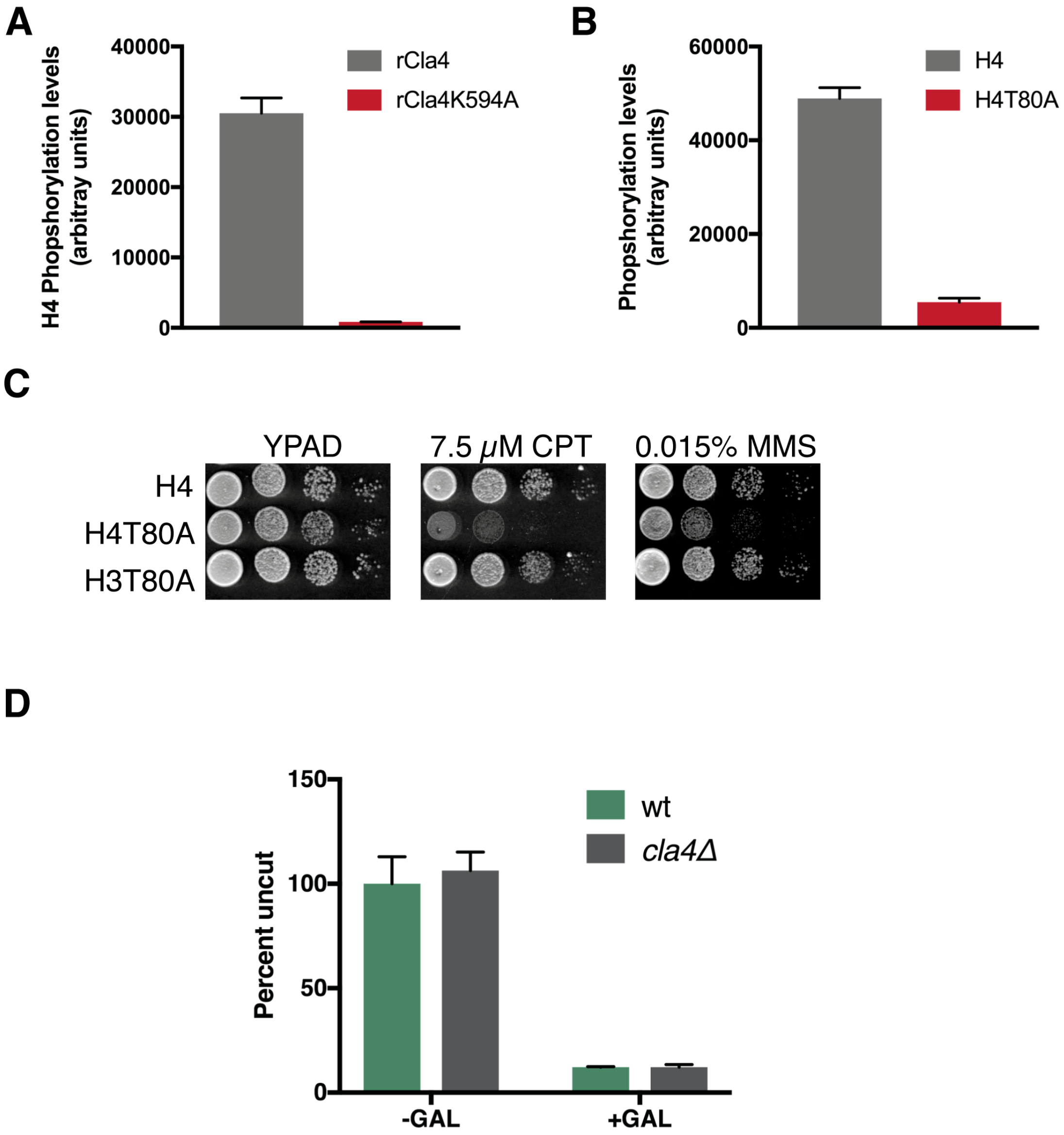


**Figure S3. Cla4 phosphorylates H4T80 *in vivo* and *in vitro*. Related to Figure 2 and Figure 3. (A)** and **(B)** Quantification of kinase experiments shown in Figure 2D and 2E respectively. Data are represented as mean +SEM of two independent experiments. **(C)** Spot test for DNA damage sensitivity of different yeast histone point mutants as indicated. 10-fold serial dilutions were used. **(D)** HO cutting efficiency was measured by qPCR using primers encompassing the HO cut site and normalized to the *PRP8* reference locus. The mean of the non-induced samples was calculated as 100% uncut. Graphs show mean +SEM of three biological replicates.

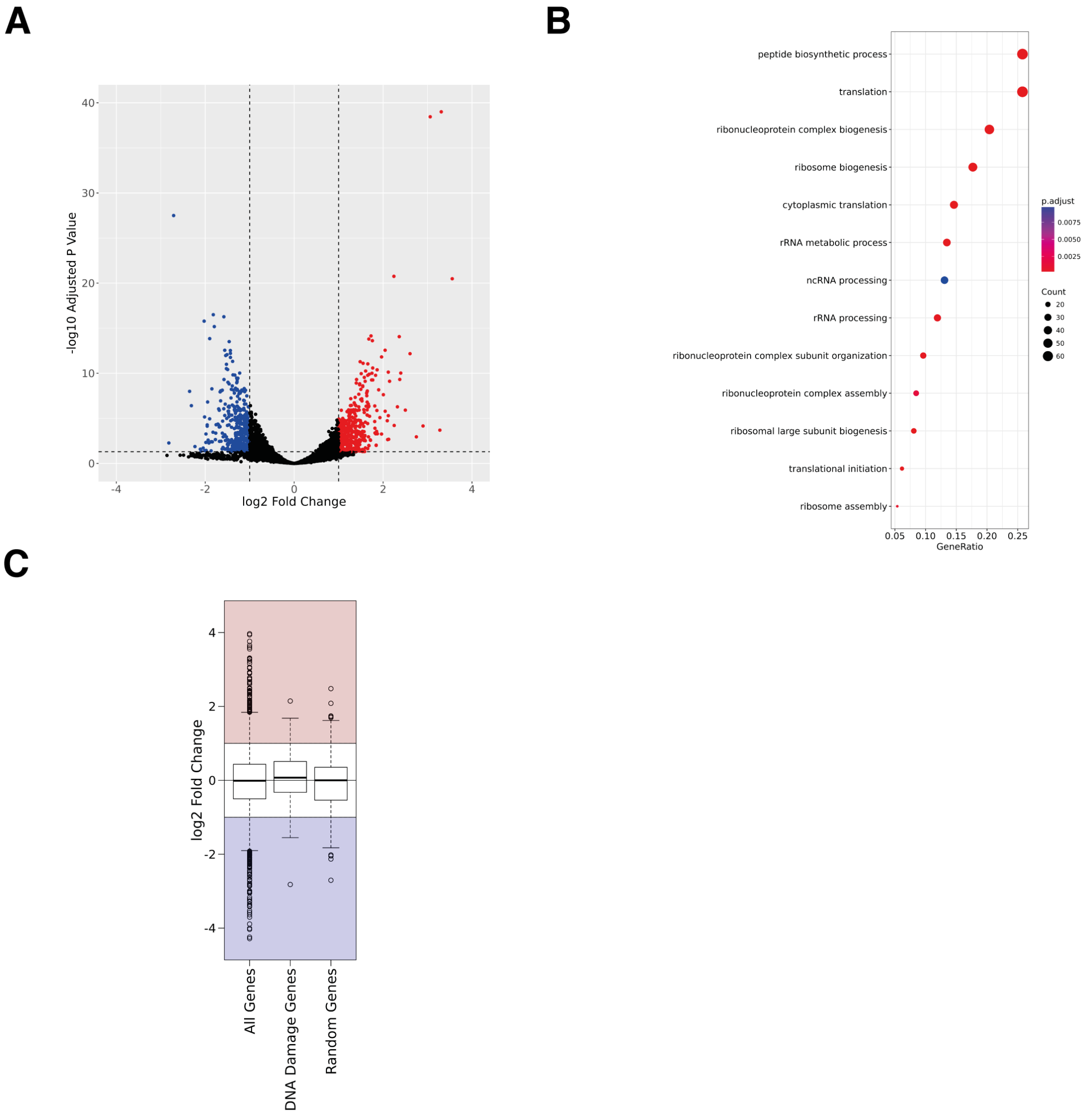


**Figure S4. H4T80A mutation does not particularly impair the expression of genes involved in the DDR. Related to Figure 4. (A)** Volcano plot showing the global differential expression between H4T80A mutant and wild type samples. The x-axis represents the log2 fold change and the y-axis represents the -log10 of the adjusted p-value from the DESeq2 differential expression analysis. Significantly up-regulated (fold change > 2, adjusted p value < 0.05) and down-regulated (fold change < 2, adjusted p value < 0.05) genes are highlighted in red and blue respectively. **(B)** Dot-plot of gene ontology terms enriched for genes down-regulated in H4T80A mutant cells compared to wild type. For each term, GeneRatio represents the proportion of genes in a given GO term that are significantly down-regulated. The size of the dots is representative of the total number of genes, whilst the colour is representative of the p value from the over-representation test. Genes up-regulated in the H4T80A mutant show no enrichment in the GO analysis. **(C)** Boxplot showing the distribution of log2 fold change values from the DESeq2 differential expression analysis for; all genes, 206 genes involved in the DNA damage response (based on GO term GO:0006974), and a random subset of 206 genes. Genes up- or down-regulated greater than 2-fold are highlighted in red or blue respectively.

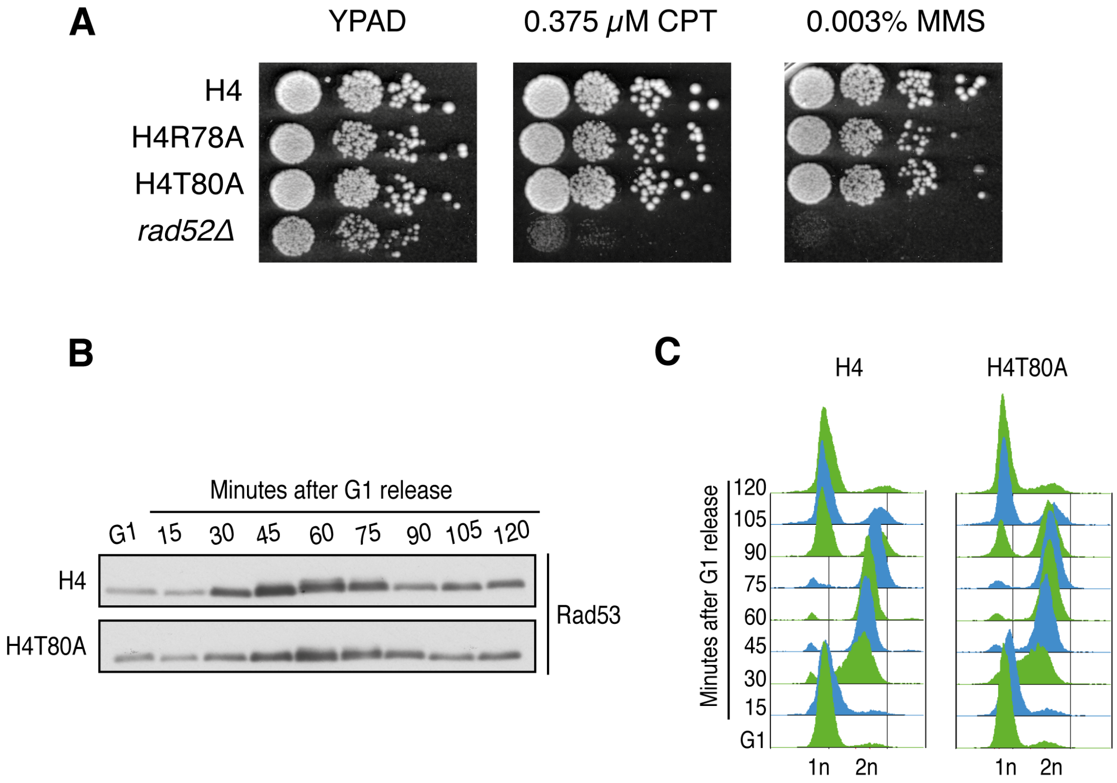


**Figure S5. H4T80A mutation does not impair cell cycle progression. Related to Figure 4.** **(A)** Spot test for DNA damage sensitivity of different yeast mutants as indicated. 10-fold serial dilutions were used. **(B)** Immunoblot analysis of Rad53 phosphorylation levels evaluated by mobility shift in SDS-PAGE gels. Wild type or H4T80A mutant cells were arrested in G1 using α-factor, and then released for 120 min. Samples were taken every 15 min. α-factor was added again after 45 min to arrest cells in G1. **(C)** Flow cytometry analysis of the same samples taken in B.

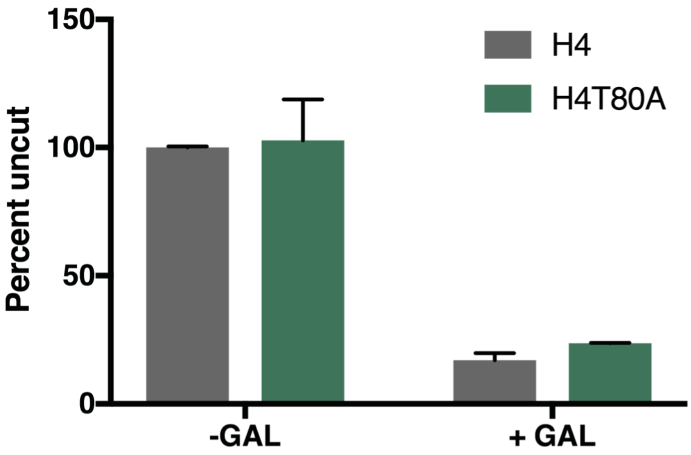


**Figure S6. DSB is induced to the same extent in H4T80A mutant and wild type cells. Related to Figure 5.** HO cutting efficiency was measured by qPCR using primers encompassing the HO cut site and normalized to the *PRP8* reference locus. The mean of the non-induced samples was calculated as 100% uncut. Graphs show mean +SEM of two biological replicates.

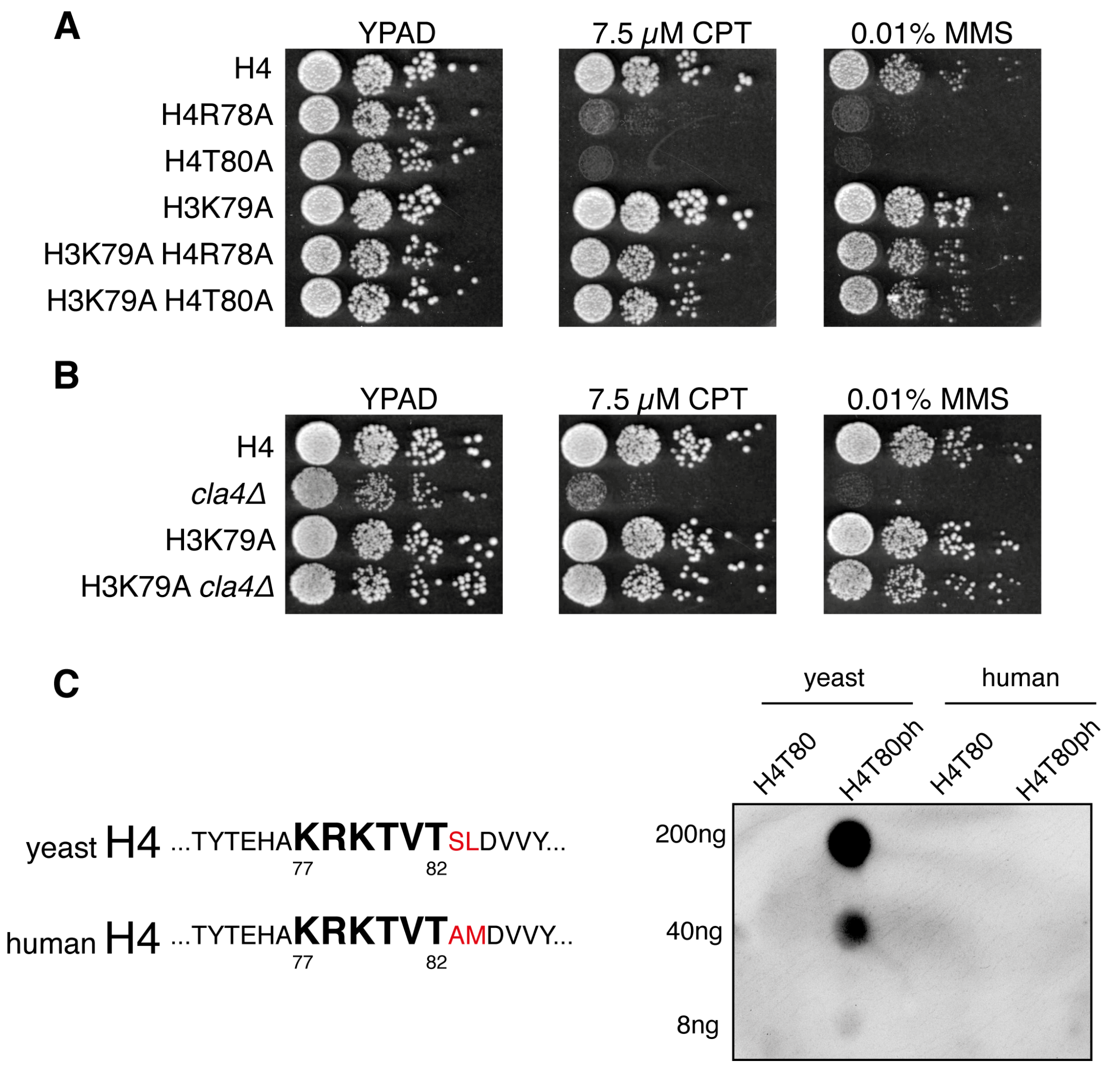


**Figure S7. Mutation of H3K79A supresses H4T80A and *cla4Δ* mutant hypersensitivity to DNA damaging agents. Related to Figure 6. (A)** and **(B)** Spot test for DNA damage sensitivity of different yeast mutants as indicated. 10-fold serial dilutions were used. **(C) Left** Comparison of yeast and human histone H4 L2 loop sequence. **Right** Dot-blot analysis using H4T80ph specific antibody. 5-fold serial dilutions of different peptides, as indicated, were spotted onto a PVDF membrane.

| Strain | Genotype | Source |
|---|---|---|
| PTY1 | *Mat a hht2,hhf2::natMX4*, *hht1,hhf1::kanMX4*<br>pURA3-*HHT1-HHF1* | (Tessarz et al., 2014) |
| GMY041 | PTY1 *cla4::hphMX4* | This study |
| GMY042 | PTY1 *skm1::hphMX4* | This study |
| GMY043 | PTY1 *ste20::hphMX4* | This study |
| GMY044 | PTY1 *rad52::hphMX4* | This study |
| GMY045 | PTY1 *RAD9-3HA::HIS3* | This study |
| GMY046 | PTY1 *RTT107-3HA::HIS3* | This study |
| GMY047 | PTY1 *dot1::HIS3* | This study |
| GMY048 | PTY1 *cla4::hphMX4*, *dot1::HIS3* | This study |
| PTY2 | *Mat a hht2,hhf2::KanMX4*, *hht1,hhf1::HIS3*,<br>*hta1,htb1::NatMX6, hta2, htb2::hphMX6*<br>pURA3-*HHT1-HHF1* | (Tessarz et al., 2014) |
| JKM179 | *MATα ade1 leu2*-3,112, *lys5, trp1::hisG, ura3*-52,<br>*hml::ADE1 hmr::ADE1 ade3::GAL::HO* | (Lee et al., 1998) |
| GMY049 | JKM179 *CLA4-6HA::kanMX4* | This study |
| GMY050 | JKM179 *cla4::hphMX4* | This study |
| GMY051 | JKM179 *hht2,hhf2::natMX4*, *hht1,hhf1::kanMX4* | This study |
| GMY052 | GMY051 *RTT107-6HA::hphMX4* | This study |
| GMY053 | *Mat a hht2,hhf2::natMX4*, *hht1,hhf1::LEU2*<br>pURA3-*HHT1-HHF1* | This study |
| GMY053 | GMY053 *rad53-R605A::kanMX6* | |
| MKY5 | *MATα ade2-1 can1-100 his3-11 leu2-3,112 trp1-1 ura3-1* | (Leung et al., 2016) |
| MKY1671 | MKY5, *rtt107::kanMX6*<br>*pRS315, RTT107-3XFLAG::natMX6* | (Leung et al., 2016) |
| MKY1672 | MKY5, *rtt107::kanMX6*<br>*pRS315, rtt107-K887M-3XFLAG::natMX6* | (Leung et al., 2016) |
| MKY1731 | MKY5, *rtt107::KANMX6*<br>*pRS315, rtt107-K426M-3XFLAG::NATMX6* | (Leung et al., 2016) |

**Table S1. Yeast Strains used in this study. Related to Key Resources Table.** Strains are derivatives of W303. Histone point mutants were shuffled into yeast by counter-selection on 5-FOA.
