# Supplementary material for: Phosphorylation of Histone H4T80 Triggers DNA Damage Checkpoint Recovery
Source: Mol Cell. 2018 Nov 15;72(4):625–635.e4. doi: 10.1016/j.molcel.2018.09.023 (PMC6242705; doi:10.1016/j.molcel.2018.09.023)
Supplement: Document S2. Article plus Supplemental Information [file mmc2.pdf]

Article

Molecular Cell

Phosphorylation of Histone H4T80 Triggers DNA Damage Checkpoint Recovery

Graphical Abstract

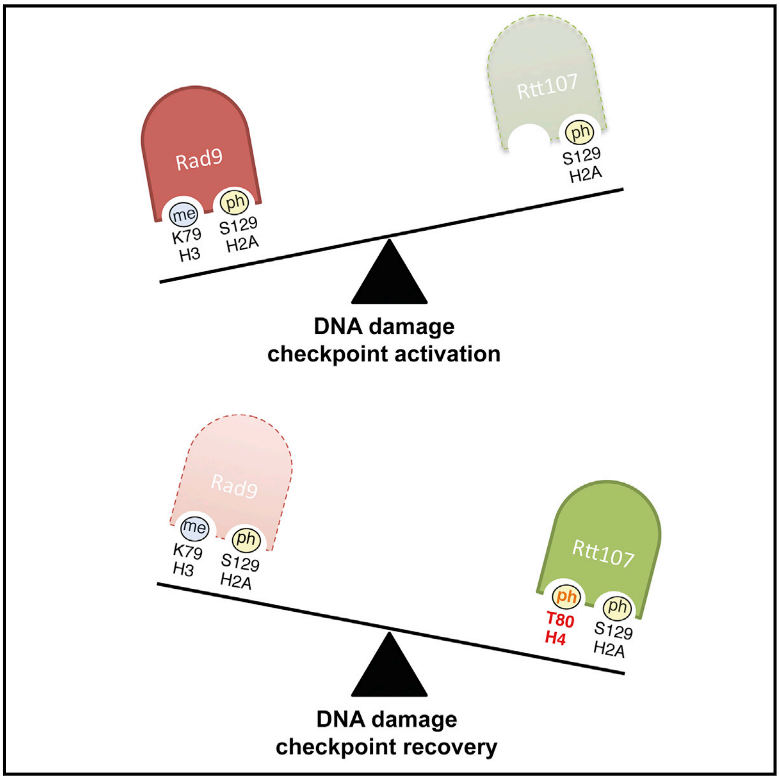

Highlights

- Histone H4T80 phosphorylation regulates DNA damage checkpoint recovery
- In response to DNA damage, PAK family kinase Cla4 phosphorylates H4T80
- H4T80 phosphorylation promotes timely recruitment of the scaffold protein Rtt107
- H4T80ph-bound Rtt107 counteracts Rad9 binding to H3K79me

Authors

Gonzalo Millan-Zambrano, Helena Santos-Rosa, Fabio Puddu, Samuel C. Robson, Stephen P. Jackson, Tony Kouzarides

Correspondence

gmillan@us.es (G.M.-Z.), t.kouzarides@gurdon.cam.ac.uk (T.K.)

In Brief

Termination of the checkpoint-signaling cascade is essential for resumption of normal cell growth after DNA damage. Millan-Zambrano et al. show that phosphorylation of histone H4T80 regulates the transition between DNA damage checkpoint activation and recovery by promoting timely recruitment of Rtt107 protein.

Millan-Zambrano et al., 2018, Molecular Cell *72*, 625–635
November 15, 2018 © 2018 The Authors. Published by Elsevier Inc.
https://doi.org/10.1016/j.molcel.2018.09.023

CellPress

Phosphorylation of Histone H4T80 Triggers DNA Damage Checkpoint Recovery

Gonzalo Millan-Zambrano,[1,4,*] Helena Santos-Rosa,[1,4] Fabio Puddu,[2] Samuel C. Robson,[3] Stephen P. Jackson,[2] and Tony Kouzarides[1,5,*]
[1]The Wellcome Trust/Cancer Research UK Gurdon Institute and Department of Pathology, University of Cambridge, Tennis Court Road, Cambridge CB2 1QN, UK
[2]The Wellcome Trust/Cancer Research UK Gurdon Institute and Department of Biochemistry, University of Cambridge, Tennis Court Road, Cambridge CB2 1QN, UK
[3]School of Pharmacy and Biomedical Science, University of Portsmouth, White Swan Road, Portsmouth PO1 2DT, UK
[4]These authors contributed equally
[5]Lead Contact
*Correspondence: gmillan@us.es (G.M.-Z.), t.kouzarides@gurdon.cam.ac.uk (T.K.)
https://doi.org/10.1016/j.molcel.2018.09.023

SUMMARY

In response to genotoxic stress, cells activate a signaling cascade known as the DNA damage checkpoint (DDC) that leads to a temporary cell cycle arrest and activation of DNA repair mechanisms. Because persistent DDC activation compromises cell viability, this process must be tightly regulated. However, despite its importance, the mechanisms regulating DDC recovery are not completely understood. Here, we identify a DNA-damage-regulated histone modification in *Saccharomyces cerevisiae*, phosphorylation of H4 threonine 80 (H4T80ph), and show that it triggers checkpoint inactivation. H4T80ph is critical for cell survival to DNA damage, and its absence causes impaired DDC recovery and persistent cell cycle arrest. We show that, in response to genotoxic stress, p21-activated kinase Cla4 phosphorylates H4T80 to recruit Rtt107 to sites of DNA damage. Rtt107 displaces the checkpoint adaptor Rad9, thereby interrupting the checkpoint-signaling cascade. Collectively, our results indicate that H4T80ph regulates DDC recovery.

INTRODUCTION

Genome integrity is continuously threatened by DNA damage arising from both exogenous and endogenous sources. However, the DNA damage response (DDR) pathway identifies and repairs damaged DNA to ensure that the genetic information is faithfully maintained. The eukaryotic genome is compacted into chromatin, whose fundamental repeating unit is the nucleosome. Nucleosomes consist of 147 base pairs of DNA tightly wrapped around a core histone octamer, which is composed of two copies of histones H2A, H2B, H3, and H4 (Luger et al., 1997). Importantly, chromatin structure regulates all DNA-based processes, including the DDR. In this regard, histones are subject to post-translational modifications that change chromatin structure and provide docking sites for other proteins. These modifications are dynamically deposited and removed by chromatin-modifying enzymes in a tightly regulated manner (Bannister and Kouzarides, 2011).

Histone post-translational modifications occur both in the tails and the core domains. One of the first-studied core modifications was methylation of histone H3 lysine 79 (H3K79me), which plays an important role in the DDR (Giannattasio et al., 2005; van Leeuwen et al., 2002). Since then, several novel histone modifications have been identified by mass spectrometry, with many of them being localized to the core domains. However, in contrast to those present in the tails, modifications in the core region of the nucleosome are far less characterized.

In response to DNA damage, cells activate a signal transduction cascade referred to as the DNA damage checkpoint (DDC), which results in a temporary cell cycle arrest and activation of DNA repair pathways (Branzei and Foiani, 2009). The molecular mechanism regulating this signaling cascade was initially described in yeast, and later, it was shown to be conserved in mammals (Elledge, 1996; Harper and Elledge, 2007). In *Saccharomyces cerevisiae*, Mec1 kinase orchestrates the DDC signaling process. Mec1 is first recruited to single-stranded DNA (ssDNA) through an interaction with replication protein A (Nakada et al., 2005; Zou and Elledge, 2003) and then activated by two independent factors, Dpb11 and PCNA-like complex (Navadgi-Patil and Burgers, 2009; Puddu et al., 2011). Two direct targets of Mec1 are histone H2A serine 129 (γH2A) and the checkpoint adaptor Rad9 (Downs et al., 2000; Gilbert et al., 2001), which is recruited to sites of DNA damage via two different pathways: one relying on Dpb11 and the other one on H3K79me and γH2A (Pfander and Diffley, 2011; Puddu et al., 2008). Rad9 plays a crucial role in the signaling cascade, working as an adaptor between Mec1 and its target kinase Rad53, whose activation is essential for the coordination of the DDR (Pellicioli and Foiani, 2005). Once fully activated, Rad53 is released from the Rad9 complex, leading to an amplification of the checkpoint signal (Branzei and Foiani, 2006).

Whereas the molecular mechanisms regulating DDC activation are well understood, comparatively less is known about how DDC recovery is initiated and controlled. Even though

This is an open access article under the CC BY license (http://creativecommons.org/licenses/by/4.0/).

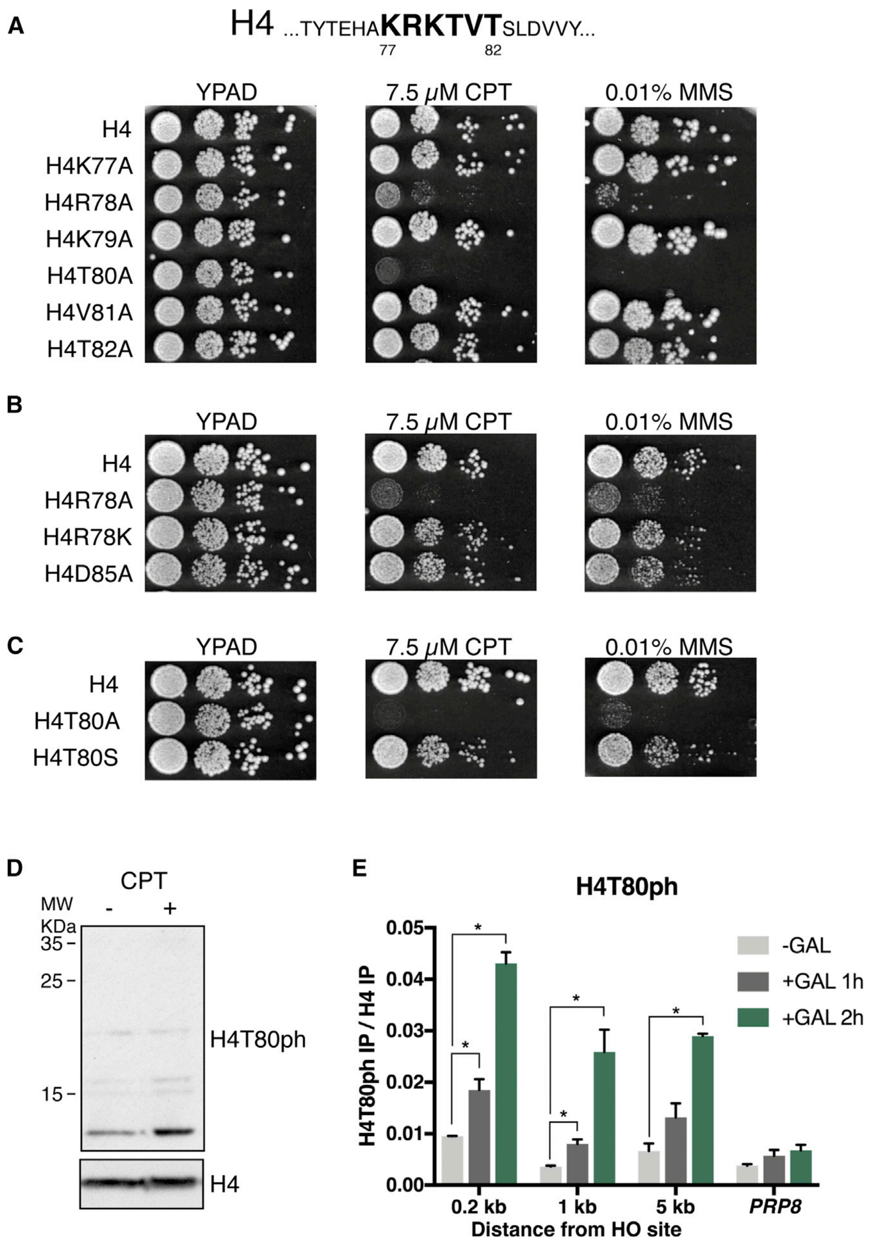

Figure 1. Histone H4T80ph Promotes Cell Survival in Response to DNA Damage

(A–C) (A, upper) Sequence of yeast histone H4 L2 loop. (A, lower; B and C) Spot test for DNA damage sensitivity of yeast cells harboring different histone H4 point mutations as indicated. 10-fold serial dilutions were used.

(D) Immunoblot analysis of purified yeast histones. Cells were arrested in G1 using α factor and then released in the absence or presence of 20 μM CPT for 45 min. Purified yeast histones were separated by SDS-PAGE in 17% acrylamide gels. Blots were probed with anti-H4T80ph antibody and then re-probed with anti-H4 antibody as indicated.

(E) ChIP-qPCR experiments showing H4T80ph recruitment to the DSB site after galactose induction. *PRP8* locus was used as a negative control. Data are represented as mean + SEM of three biological replicates (*p < 0.05; t test).

different factors have been shown to be involved in the process (Chen et al., 2008; Keogh et al., 2006; Shimada et al., 2008; Vaze et al., 2002), the molecular mechanisms governing DDC recovery are still not apparent. However, this is a critically important process because persistent checkpoint activation is detrimental to cell viability (Clerici et al., 2001). Termination of the DDC requires inactivation of the downstream kinase Rad53 to allow resumption of the cell cycle. Although direct inactivation of Rad53 kinase can be achieved by the action of specific phosphatases (Leroy et al., 2003; O'Neill et al., 2007), full DDC recovery requires interruption of the upstream signaling cascade. In this context, it has been proposed that the Rtt107-Slx4 protein complex can outcompete Rad9 from γH2A and Dpb11 (Cussiol et al., 2015; Ohouo et al., 2013). However, the mechanism regulating this transition still remains elusive. The findings presented here identify H4T80ph, a DNA-damage-regulated histone core modification, as a key regulator of the switch between DDC activation and DDC recovery.

RESULTS

H4T80ph Promotes Cell Survival in Response to DNA Damage

Within nucleosomes, the lateral surface of the core histone octamer is in direct contact with the DNA and is of particular interest, because it is fundamental to nucleosome integrity and therefore has the potential to affect all DNA-based processes (Lawrence et al., 2016). In this regard, the amino acid residues within the L2 loop of histone H4 are particularly noteworthy, because they contact both DNA and the L1 loop of histone H3 (Luger et al., 1997). In order to shed some light on the function(s) of this specific region, we individually mutated every residue in the L2 loop of H4 (residues 77–82; Figure 1A) to alanine in *Saccharomyces cerevisiae* and investigated phenotypes of resulting strains. We observed that, in contrast to other mutations analyzed, mutation of either arginine 78 (H4R78A) or threonine 80 (H4T80A) results in severe hypersensitivity to the DNA-damaging agents camptothecin (CPT) and methyl methanesulfonate (MMS) (Figure 1A).

We initially focused on the possible role of H4R78 in the DDR. H4R78 forms hydrogen bonds with histone H4 aspartate 85 (H4D85) (Figure S1A), but disruption of this salt bridge by H4D85A mutation does not elicit DNA damage hypersensitivity (Figure 1B), suggesting that this interaction is not necessary for the DDR. We then asked whether methylation of H4R78 might be required for DNA damage resistance. However, mutation of

H4R78 to lysine does not cause DNA damage hypersensitivity (Figure 1B), indicating that the presence of a basic amino acid residue at position 78 is sufficient for cell viability in response to DNA damage.

Although H4T80 could exert its functions in various ways, we were intrigued by the possibility that it might be phosphorylated in a manner requiring a basic amino acid residue at position 78. In this regard, certain basophilic protein kinases are known to target either threonine or serine preceded by basic residues (Mok et al., 2010). In agreement with the hypothesis that H4T80 phosphorylation may be required for DNA damage resistance, mutation of this residue to serine, which can also be substrate of basophilic kinases, largely rescues the DNA damage hypersensitivity of the H4T80A mutants (Figure 1C). Interestingly, mutation of H4T80 to either aspartic acid or glutamic acid, which can mimic a permanent phosphorylated state, causes cell death (Figure S1B). Collectively, these results suggest that phosphorylation of H4T80 is important for cell survival in response to DNA damage.

Different large-scale mass spectrometry studies identified histone H4T80ph in higher eukaryotes (Bennetzen et al., 2010; Hornbeck et al., 2015; Lundby et al., 2012; Mertins et al., 2014; Olsen et al., 2010; Parker et al., 2015; Tsai et al., 2015). However, its function still remains completely unexplored. To investigate the possible role of H4T80 phosphorylation in the DDR, we raised an antibody against H4T80ph, whose specificity was first assessed by peptide dot-blot analysis. This antibody specifically recognizes H4T80ph peptides, but not other H4 phospho-peptides, such as H4T30ph or H4T96ph (Figure S2A). Moreover, the antibody does not recognize histone H3T80ph peptides, in which the phosphorylated threonine is also preceded by a basic residue (Figure S2B). Immunoblot studies using purified yeast histones (Figure S2C) showed that the antibody reacts with H4 and that the signal is strongly reduced upon phosphatase treatment (Figure S2D), indicating that it recognizes phosphorylated H4. To determine whether it specifically reacts with H4T80ph, we isolated histones from H4T80A mutant cells. In contrast to wild-type histone H4, the antibody does not detect mutant histone H4T80A (Figure S2E). Together, these results therefore confirm that the antibody specifically recognizes H4T80ph in yeast.

Because the H4T80A mutant is sensitive to DNA-damaging agents, we asked whether H4T80ph levels might be regulated in response to DNA damage. Importantly, immunoblot studies confirmed that H4T80ph levels increase upon CPT treatment (Figure 1D). To investigate whether H4T80ph occurs at sites of DNA damage, we used a well-established experimental system in which addition of galactose induces a single, persistent DNA double-strand break (DSB) (Figure S2F; Lee et al., 1998). This system has been widely used before to monitor the recruitment of DNA repair factors and chromatin regulators to a DSB by chromatin immunoprecipitation (ChIP) (Bennett et al., 2013; Chen et al., 2008, 2012; Lee et al., 2014; Sugawara et al., 2003; Wang and Haber, 2004). Upon galactose induction, H4T80ph levels increase at the DSB site (Figure 1E), but not in an unrelated region, suggesting that H4T80ph is particularly accumulated at sites of DNA damage. Collectively, the above findings support that H4T80 phosphorylation promotes cell survival in response to DNA damage.

PAK Family Kinase Cla4 Phosphorylates Histone H4T80

We next sought to identify the kinase(s) responsible for H4T80ph. Although yeast basophilic kinases commonly show a strong selectivity for arginine residues three amino acid residues upstream of the phosphorylation site (P-3), only four of them (Ipl1, Cla4, Ste20, and Skm1) are selective for arginine at the P-2 position (Mok et al., 2010). As the role of Ipl1 in the regulation of chromosome condensation via phosphorylation of H3, but not H4, has been extensively characterized (Hsu et al., 2000), we focused on the other three kinases, which are members of the p21-activated kinase (PAK) family (Zhao and Manser, 2012). Cla4 is involved in septin ring assembly, actin polymerization, and cytokinesis (Cvrcková et al., 1995; Versele and Thorner, 2004). Ste20 was first identified as an essential protein in the mating pathway (Leberer et al., 1992), although it also plays some overlapping functions with Cla4 (Cvrcková et al., 1995). Finally, less is known about the functions of Skm1, which is a paralog of Cla4 (Martín et al., 1997). Notably, none of these three yeast kinases have been specifically linked to the DDR.

Deletion of *CLA4*, but not *SKM1* or *STE20*, results in DNA damage hypersensitivity (Figure 2A), suggesting that Cla4 is involved in the DDR. Moreover, immunoblot analysis showed that *cla4Δ* mutant cells exhibit a strong reduction in H4T80ph levels compared to wild-type cells (Figure 2B), indicating that Cla4 is required for H4T80 phosphorylation *in vivo*. To address whether Cla4 can directly phosphorylate H4 *in vitro*, we produced recombinant wild-type Cla4 (rCla4) and a kinase dead version (rCla4K594A; Figure 2C; Versele and Thorner, 2004). Importantly, wild-type Cla4, but not the kinase dead version, phosphorylates H4 in a mixture of free histones *in vitro* (Figure 2D). To determine whether Cla4 phosphorylates H4T80, we used a histone H4 peptide encompassing the region (residues 70–90). Notably, rCla4 phosphorylates histone H4T80 peptide, but not a version of this peptide in which T80 is replaced by alanine (Figure 2E). Taken together, these results indicate that Cla4 is responsible for H4T80 phosphorylation.

Our *in vitro* assays revealed some Cla4 activity, although to a much lesser extent, toward other histones. In line with this, it has been reported that H3 is phosphorylated at an equivalent position (H3T80) in mammalian cells (Hammond et al., 2014). H3T80 is also preceded by a basic residue, although at the P-1 position, raising the possibility that Cla4 could be responsible for H3T80ph. However, in contrast to H4T80A, the H3T80A mutant does not display hypersensitivity to DNA damaging agents (Figure S3C), further supporting the notion that Cla4 specifically phosphorylates H4T80 to promote cell survival in response to DNA damage.

To explore whether Cla4 phosphorylates H4T80 at sites of DNA damage, we first analyzed the DSB recruitment of Cla4 kinase by ChIP. Upon galactose induction, Cla4 is recruited to the DSB site (Figure 3A), suggesting that it plays a direct role in the DDR. Moreover, ChIP analysis revealed that H4T80ph accumulation at the DSB site is dependent on Cla4 (Figure 3B). Importantly, defective accumulation of H4T80ph in *cla4Δ* mutant cells is not due to impaired DSB induction (Figure S3D). Together, these results indicate that Cla4 is responsible for H4T80 phosphorylation at sites of DNA damage.

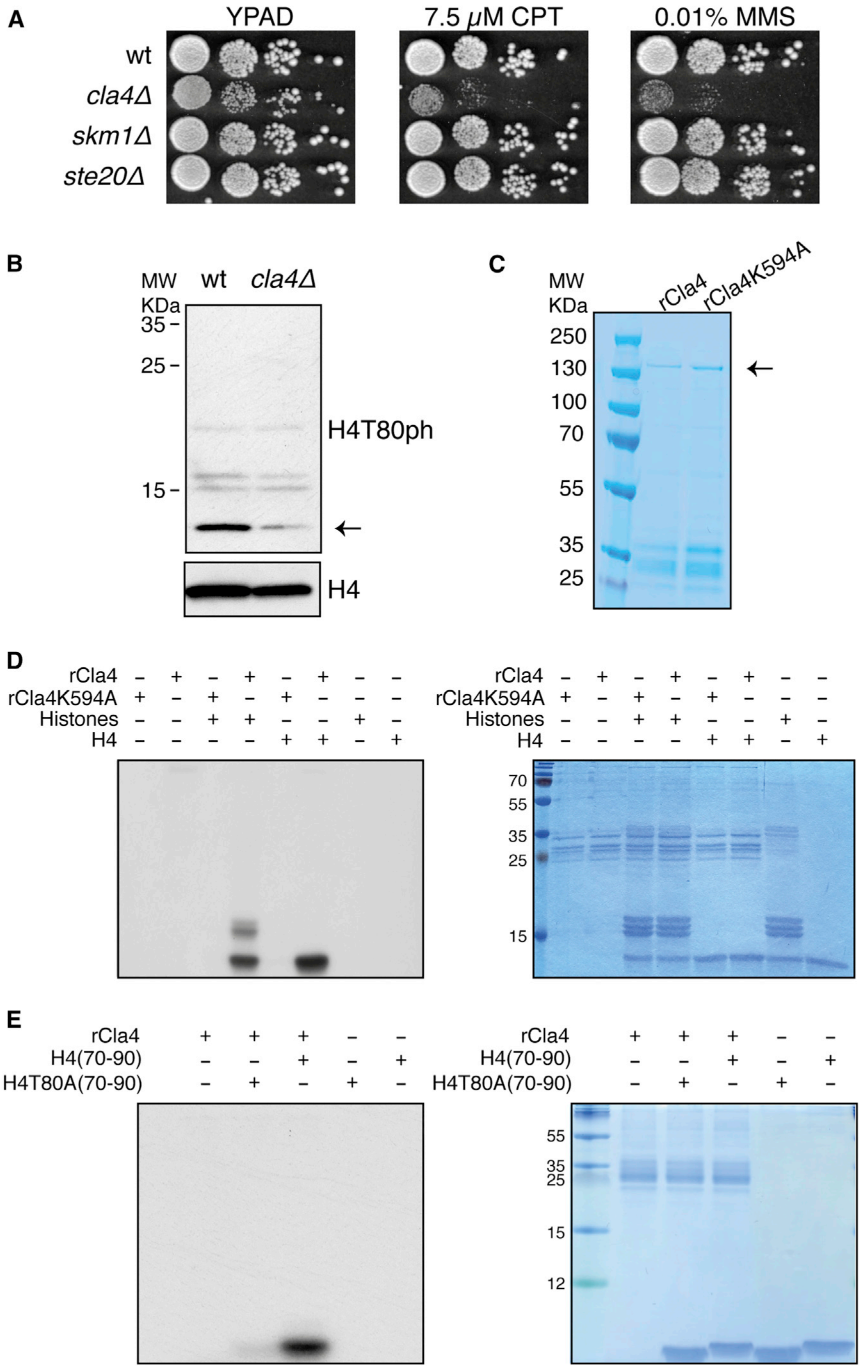

Figure 2. PAK Family Kinase Cla4 Phosphorylates Histone H4T80

(A) Spot test for DNA damage sensitivity of different kinase deletion mutants as indicated. 10-fold serial dilutions were used.

(B) Immunoblot analysis of purified yeast histones. Both wild-type and *cla4Δ* mutant cells were grown to exponential phase. Purified yeast histones were separated by SDS-PAGE in 17% acrylamide gels. Blots were probed with anti-H4T80ph antibody and then re-probed with an anti-H4 antibody as indicated. ← corresponds to histone H4.

(C) Coomassie-blue-stained gel showing purified wild-type glutathione S-transferase (GST)-Cla4 (rCla4) and the corresponding catalytically inactive mutant (rCla4K594A). ← corresponds to GST-Cla4.

(D) *In vitro* kinase assay using γ^{32}P- ATP. Both recombinant wild-type Cla4 (rCla4) and a kinase inactive version (rCla4K594A) were incubated with either purified core histones or purified histone H4. Reactions were separated by SDS-PAGE in 17% acrylamide gels. Right panel shows Coomassie blue stained gel, and left panel shows the corresponding autoradiogram.

(E) *In vitro* kinase assay using γ^{32}P- ATP. rCla4 was incubated with a peptide encompassing Thr-80 (H4) or a version of it in which this residue was replaced by alanine (H4T80A). Reactions were separated in 17% acrylamide tricine gels. Right panel shows Coomassie blue stained gel, and left panel shows the corresponding autoradiogram.

H4T80ph Regulates the DNA Damage Checkpoint

To examine functions of H4T80ph, we next investigated the origin of the DNA damage hypersensitivity of the H4T80A mutant strain. We initially excluded the possibility that this phenotype was due to impaired expression of DDR genes by analyzing changes in gene expression profiles between wild-type and H4T80A mutant cells (Figure S4A). Importantly, genes exhibiting significant differential expression show no enrichment for DNA-damage-related Gene Ontology (GO) terms (Figure S4B). Moreover, analysis of 206 genes involved with cellular response to DNA damage stimulus (GO: 0006974) show almost no change in expression between H4T80A mutant and wild-type cells, particularly when contrasted with a randomly selected subset of 206 genes (Figure S4C).

We next explored whether H4T80ph was involved in DNA repair. In *Saccharomyces cerevisiae*, the major pathway for DSB repair is homologous recombination (HR), which is completely dependent on Rad52 (Symington, 2002). Repair of CPT- or MMS-induced DNA damage requires Rad52-dependent HR, and therefore, *rad52Δ* cells are extremely sensitive to these drugs (Figure S5A; note that *rad52Δ* cells are sensitive even to very low concentrations of CPT and MMS, at which H4R78A and H4T80A are not). If the DNA damage hypersensitivity of the H4T80A mutant was due to HR-mediated DNA repair defects, then mutation of H4T80A should not enhance *rad52Δ* phenotype. However, mutation of either H4R78A or H4T80A exacerbates the DNA damage hypersensitivity of *rad52Δ* cells (Figure 4A). Consistent with this, global genetic interaction studies have shown that deletion of *CLA4* exacerbates the DNA damage

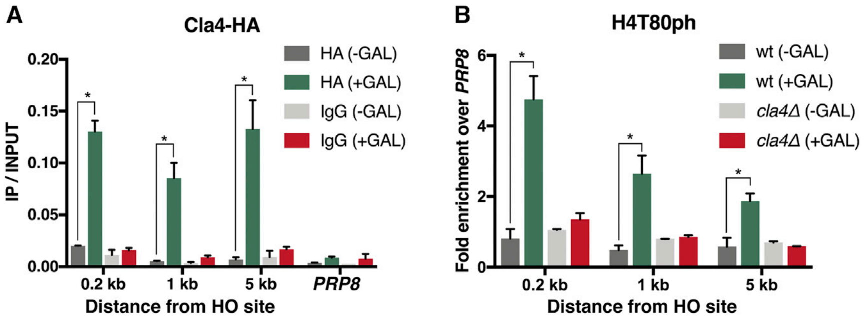

Figure 3. Cla4 Phosphorylates H4T80 at Sites of DNA Damage

(A) ChIP-qPCR experiments showing Cla4-hemagglutinin (HA) recruitment to the DSB site before and after 2 hr of galactose induction. *PRP8* locus was used as a negative control. Graphs show mean + SEM of two biological replicates (*p < 0.05; t test).

(B) ChIP-qPCR experiments showing H4T80ph enrichment at the DSB site after 2 hr of galactose induction in the indicated strains. Enrichment at the target loci were normalized to the *PRP8* reference locus. Data are represented as mean + SEM of three biological replicates (*p < 0.05; t test).

hypersensitivity of most mutants in the HR pathway, including *rad52Δ* (Bandyopadhyay et al., 2010; St Onge et al., 2007). It is therefore unlikely that the DNA damage hypersensitivity caused by the lack of H4T80ph is due to HR-mediated DNA repair defects per se.

Accordingly, we assessed whether H4T80ph was involved in activation of the DDC, a signaling cascade initiated by the recognition of ssDNA by the apical Mec1 kinase and culminating in Mec1-dependent activation of the downstream kinase Rad53 (Branzei and Foiani, 2009). To do so, we analyzed Rad53 phosphorylation levels upon CPT treatment. Both wild-type and H4T80A cells were synchronized in G1 using α factor and then released into S phase in the presence of CPT. H4T80A mutant cells exhibit stronger and prolonged activation of Rad53 compared to wild-type cells (Figure 4B), which is associated with a prolonged G2/M cell cycle arrest (Figure 4C; note that, unlike wild-type cells, H4T80A cells did not progress to G1 during the course of this analysis). Importantly, none of these phenotypes are detectable in the absence of genotoxic stress (Figures S5B and S5C). Notably, phosphorylation of histone H2A serine 129 (γH2A), which is also substrate of Mec1 (Downs et al., 2000), is not increased but actually decreased in H4T80A mutant cells (Figure 4B), suggesting that Rad53 hyperactivation observed in the H4T80A mutant is not caused by increased DNA-damage-induced Mec1 signaling. Mutation of H2AS129A abolishes γH2A and results in DNA damage hypersensitivity (Downs et al., 2000). Therefore, we asked whether defective γH2A might be the origin of the DNA damage hypersensitivity of H4T80A cells. However, mutation of H4T80A exacerbates the DNA damage hypersensitivity of H2AS129A mutant cells (Figure 4D), supporting the notion that H4T80A mutant phenotype is not merely caused by defects in H2A phosphorylation.

We next explored whether improper regulation of Rad53 activation could be responsible for H4T80A mutant phenotype. To do so, we used a hypomorphic allele of *RAD53* (*rad53-R605A*) that has been previously shown to lower Rad53 activation levels (Jablonowski et al., 2015; Ohouo et al., 2013). This allele harbors a point mutation in the FHA2 domain, which binds phosphorylated Rad9 to mediate Rad53 activation. Strikingly, the DNA damage hypersensitivity of the H4T80A mutant can be rescued by the hypomorphic allele of *RAD53* (*rad53-R605A*; Figure 4E), confirming that H4T80A mutant phenotype is due to improper regulation of Rad53 signaling. To determine whether Rad9 is involved in Rad53 hyperactivation observed in H4T80A mutant cells, we analyzed Rad9 phosphorylation levels upon CPT treatment. Interestingly, H4T80A mutant cells show increased levels of Rad9 phosphorylation that persist for longer periods of time compared to wild-type cells (Figure 4F). Collectively, these results indicate that H4T80ph plays an important role in regulating Rad9-dependent activation of Rad53 and that its absence leads to a persistent cell cycle arrest that compromises cell viability.

H4T80ph Controls Rtt107 Recruitment to Sites of DNA Damage

Histone post-translational modifications play major roles in recruiting Rad9 to sites of DNA damage, where it specifically binds histone H3 methylated at lysine 79 (H3K79me) via its Tudor domain and γH2A via its BRCA1 C-terminal (BRCT) domains (Giannattasio et al., 2005; Hammet et al., 2007). Interestingly, H3K79 and H4T80 are located next to each other in the nucleosome structure (Figure 5A), suggesting that H4T80 phosphorylation and/or the recruitment of a specific histone modification reader might regulate Rad9 chromatin binding. In line with this idea, it was recently shown that Rtt107 binds γH2A via its BRCT5/6 domains to outcompete Rad9 and downregulate Rad53 activation (Ohouo et al., 2013). However, the mechanism driving the transition from checkpoint activation (Rad9-γH2A complex) to inactivation (Rtt107-γH2A complex) remained elusive. We therefore explored whether H4T80ph could trigger this switch by regulating timely recruitment of Rtt107. To do so, we analyzed the DSB recruitment of Rtt107 in wild-type and H4T80A mutant cells by ChIP. Consistent with a role of H4T80ph in Rtt107 recruitment, mutation of H4T80A significantly reduces Rtt107 enrichment at the DSB site (Figure 5B). Importantly, defective recruitment of Rtt107 in H4T80A mutant cells is not due to impaired DSB induction (Figure S6). Together, these results indicate that H4T80ph promotes Rtt107 recruitment to sites of DNA damage.

Recruitment of Rtt107 to sites of DNA damage correlates with its Mec1-dependent phosphorylation (Balint et al., 2015). In agreement with the hypothesis that H4T80ph is important for Rtt107 recruitment and subsequent Rad53 inactivation, mutation of histone H4T80A impairs DNA-damage-induced Rtt107 phosphorylation and results in Rad53 hyperphosphorylation (Figure 5C). We noticed that Rtt107 protein abundance is significantly increased upon CPT treatment and that this increase is

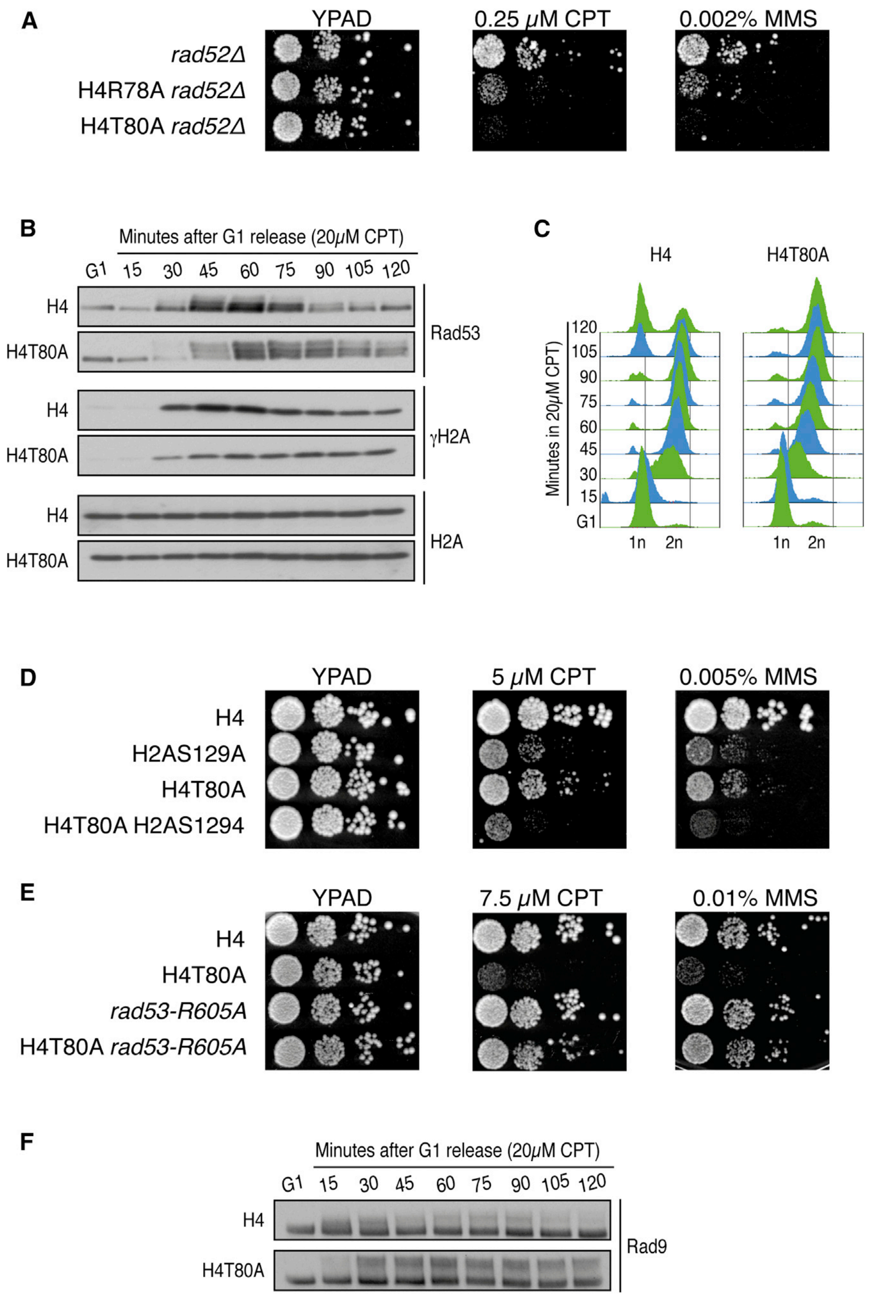

Figure 4. H4T80ph Regulates the DNA Damage Checkpoint

(A) Spot test for DNA damage sensitivity of different yeast mutants as indicated. 10-fold serial dilutions were used.

(B) Immunoblot analysis of Rad53 and histone H2A phosphorylation levels. Wild-type or H4T80A mutant cells were arrested in G1 using α factor and then released in the presence of 20 μM CPT for 120 min. Samples were taken every 15 min. α factor was added again after 45 min to arrest cells in G1. Rad53 phosphorylation was evaluated by mobility shift in SDS-PAGE gels.

(C) Flow cytometry analysis of the same samples taken in (B).

(D and E) Spot test for DNA damage sensitivity of different yeast mutants as indicated. 10-fold serial dilutions were used.

(F) Immunoblot analysis of Rad9-HA phosphorylation levels evaluated by mobility shift in NuPAGE Tris-acetate 3%–8% gel. The same experimental conditions as in (B) were used.

milder in H4T80A mutant cells compared to wild-type cells (Figure 5C). Interestingly, *RTT107* mRNA levels do not significantly change upon CPT treatment and/or H4T80A mutation (Figure 5D), indicating that differences in Rtt107 protein abundance are not due to transcriptional changes. In contrast, *RNR3* mRNA levels, which are known to be regulated by the DDC, are markedly increased upon CPT treatment, especially in the H4T80A mutant (Figure 5D). Collectively, these results suggest that Rtt107 protein may be stabilized when recruited to sites of DNA damage.

To investigate whether Rtt107 directly binds H4T80ph, we performed *in vitro* peptide pull-down experiments using yeast whole-cell extracts. Rtt107 binds to a peptide spanning histone H4T80 but only when T80 is phosphorylated (Figure 5E), further supporting the notion that H4T80ph promotes Rtt107 chromatin recruitment. Because Rtt107 contains multiple BRCT domain pairs, which commonly function as phosphoprotein-binding modules (Leung and Glover, 2011), we then questioned which one was responsible for Rtt107-H4T80ph interaction. It was recently shown by mutational analysis that Rtt107 recruitment to sites of DNA damage is mediated not only by BRCT5/6, which bind γH2A, but also by BRCT3/4, whose binding site remains unknown (Leung et al., 2016). Using Rtt107 point mutants that disrupt either BRCT3/4 or BRCT5/6 (K426M and K887M, respectively; Leung et al., 2016), we observed that Rtt107 BRCT3/4 pair, but not BRCT5/6, is absolutely required to bind H4T80ph *in vitro* (Figure 5E). Taken together, these results support a model in which histone H4T80ph promotes Rtt107 chromatin recruitment via its interaction with BRCT3/4.

H4T80ph Triggers DNA Damage Checkpoint Recovery

Our results support that, after DNA damage, Cla4 phosphorylates H4T80 to promote Rtt107 recruitment and consequent Rad53 inactivation. In this model, the absence of H4T80ph would allow prolonged Rad9 persistence on chromatin driving excessive Rad53 activation, which results in DNA damage hypersensitivity. Because H3K79 lies in close proximity to H4T80 (Figure 5A), we hypothesized that H4T80ph-bound Rtt107 could directly counteract Rad9 binding to H3K79me. In line with this

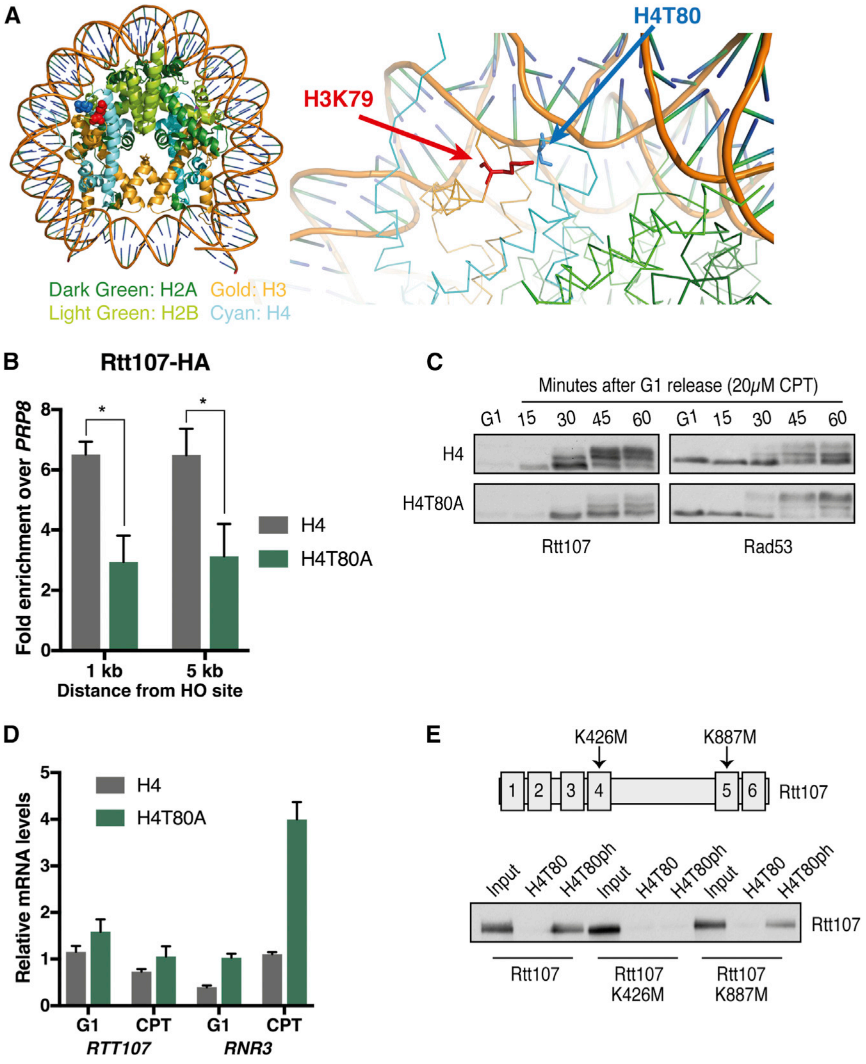

Figure 5. H4T80ph Controls Rtt107 Recruitment to Sites of DNA Damage

(A) Ribbon structure of H3 L1 loop and H4 L2 loop showing that the side chains of H4T80 and H3K79 are in close proximity.

(B) ChIP-qPCR experiments showing Rtt107-HA recruitment to regions near the DSB after 2 hr of galactose induction. Enrichment at the target loci were normalized to the *PRP8* reference locus. Data are represented as mean + SEM of three biological replicates (*$p < 0.05$; t test).

(C) Immunoblot analysis of Rtt107-HA and Rad53 phosphorylation levels evaluated by mobility shift in SDS-PAGE gels. Wild-type or H4T80A mutant cells were arrested in G1 using α factor and then released in the presence of 20 μM CPT for 60 min. Samples were taken every 15 min.

(D) RT-qPCR experiments showing *RTT107* and *RNR3* mRNA levels before and after CPT treatment. Data were normalized to *ACT1* and represent mean + SEM of three biological replicates.

(E) A schematic of the Rtt107 protein is shown (upper panel). Point mutations disrupting BRCT3/4 and BRCT5/6 pairs, respectively, are shown. The lower panel shows an immunoblot analysis of Rtt107-FLAG *in vitro* peptide binding assays. Wild-type and Rtt107 mutant cells were grown to exponential phase. Whole-cell extracts were then prepared and incubated with immobilized peptides as indicated.

hypothesis, deletion of *DOT1*, the only H3K79 methyltransferase, suppresses the DNA damage hypersensitivity of H4T80A and *cla4Δ* mutant cells (Figures 6A and 6B). Moreover, similar results were obtained by mutating H3K79A (Figures S7A and S7B), highlighting the specific crosstalk between H3K79me and H4T80ph. Together, these results further support the notion that H4T80ph plays an important role in regulating Rad9-dependent activation of Rad53.

Because depletion of H3K79me suppresses the DNA damage hypersensitivity of H4T80A mutant cells, we asked whether it might also restore the DSB recruitment of Rtt107. However, H3K79A H4T80A double-mutant cells show a similar defect in Rtt107 recruitment compared to H4T80A mutant cells (Figure 6C), indicating that, even in the absence of H3K79me, H4T80ph is still necessary to properly recruit Rtt107 to sites of DNA damage. Therefore, we next investigated whether depletion of H3K79me suppressed Rad53 hyperactivation observed in H4T80A mutant cells. Upon CPT treatment, H4T80A mutant cells exhibit defective Rtt107 phosphorylation, along with stronger and prolonged phosphorylation of Rad53, compared to wild-type cells (Figure 6D). Surprisingly, *DOT1* deletion partially rescues defective Rtt107 phosphorylation in H4T80A mutant cells (Figure 6D), suggesting that reduced Rad9 binding may favor increased Rtt107 recruitment. However, we did not observe a similar behavior by ChIP, indicating that Rtt107 cannot be stably recruited in the absence of H4T80ph. Most importantly, we found that deletion of *DOT1* suppresses Rad53 hyperphosphorylation in H4T80A cells (Figure 6D), further suggesting that this phenotype is due to Rad9 persistence on chromatin. Collectively, these results support the notion that, in response to DNA damage, Rtt107 and Rad9 bind to the same nucleosome core region in order to regulate Rad53 activation. It is important to note that Rtt107 protein upregulation upon CPT treatment is transient (Figure 6D), suggesting that there is a relatively narrow window of opportunity for it to be recruited to sites of DNA damage. Notably, we observed that H4T80A mutant cells downregulate Rtt107 protein levels despite the fact that Rad53 has not been inactivated yet (Figure 6D), critically missing this time window. Therefore, we conclude that H4T80ph is crucial for timely Rtt107-dependent downregulation of Rad53 activity.

DISCUSSION

Reversal of the DDC not only requires efficient repair of DNA lesions but also termination of the checkpoint signaling to allow

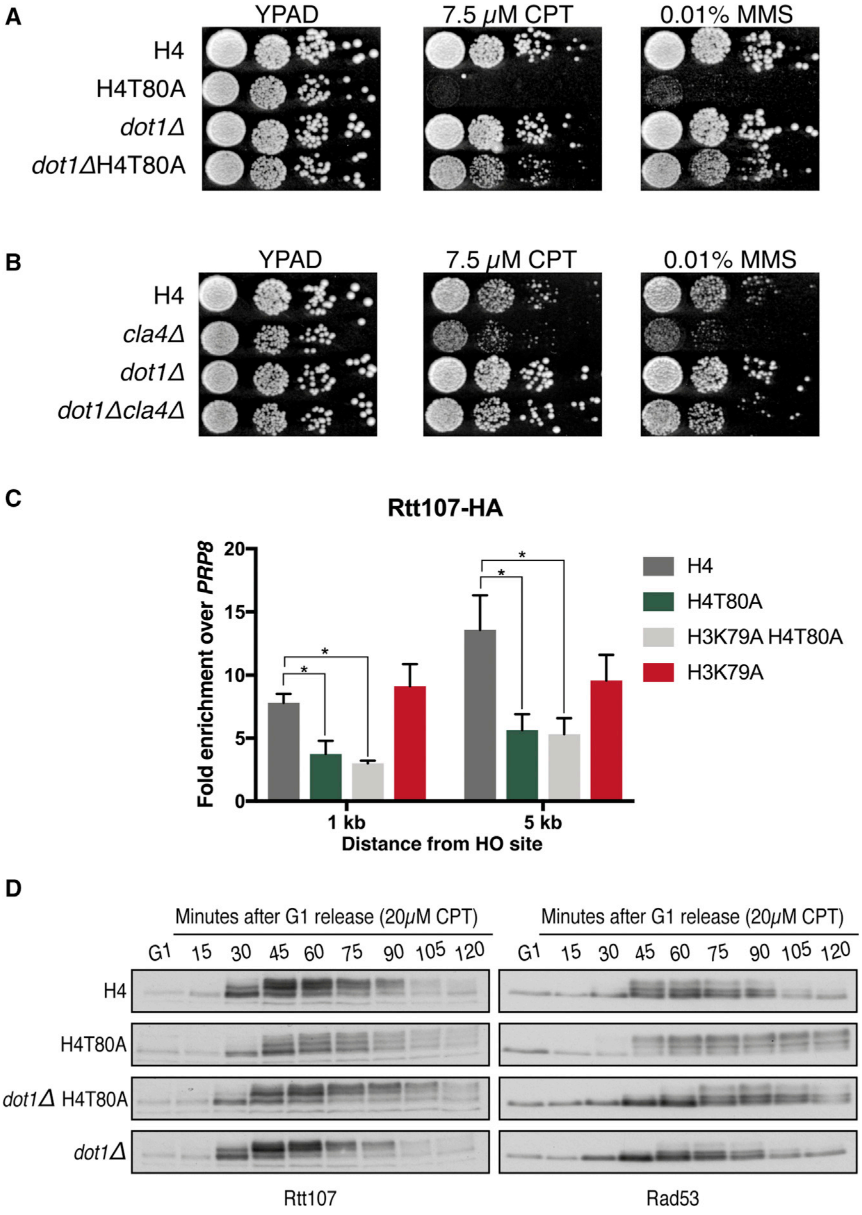

Figure 6. H4T80ph Triggers DNA Damage Checkpoint Recovery

(A and B) Spot test for DNA damage sensitivity of different yeast mutants as indicated. 10-fold serial dilutions were used.

(C) ChIP-qPCR experiments showing Rtt107-HA recruitment to regions near the DSB after 2 hr of galactose induction in the indicated strains. Enrichment at the target loci were normalized to the *PRP8* reference locus. Data are represented as mean + SEM of three biological replicates (*$p < 0.05$; t test).

(D) Immunoblot analysis of Rtt107-HA and Rad53 phosphorylation levels evaluated by mobility shift in SDS-PAGE gels. Indicated strains were arrested in G1 using α factor and then released in the presence of 20 μM CPT for 120 min. Samples were taken every 15 min.

resumption of the cell cycle. Whereas the molecular mechanisms regulating DDC activation are well characterized, our understanding of how DDC recovery is initiated and controlled is still limited. Chromatin structure regulates all DNA-based processes, including the DNA damage response, and it is well established that histone post-translational modifications, such as H3K79me and γH2A, play major roles in DDC activation (Giannattasio et al., 2005; Hammet et al., 2007). Given the toxicity of persistent DDC activation, it seems therefore surprising that no DDC recovery-dedicated histone post-translational modification had been discovered so far. Our work identifies H4T80ph, a DNA-damage-induced histone modification, as an important regulator of recovery from DNA damage.

H4T80ph is important for cell survival to DNA damage. Its absence causes prolonged Rad9-dependent activation of Rad53 and persistent cell cycle arrest. In particular, we show that H4T80ph is required for Rtt107-dependent inactivation of Rad53. It was proposed that Rtt107 prevents excessive Rad9-dependent Rad53 activation by binding to γH2A (Ohouo et al., 2013). However, in this scenario, both proteins would compete for the same binding site, and therefore, the mechanism triggering the switch between checkpoint activation and recovery would not be apparent. Moreover, it was not clear how H3K79me-bound Rad9 would be outcompeted. Our findings support a model in which DNA-damage-dependent phosphorylation of H4T80 determines timely recruitment of Rtt107, the consequent displacement of Rad9, and the interruption of the checkpoint-signaling cascade, leading to resumption of normal cell growth (Figure 7).

Cla4, the kinase responsible for this histone modification, plays key roles in promoting assembly of the septin ring at the bud neck to regulate polarized growth and cytokinesis (Cvrcková et al., 1995; Versele and Thorner, 2004). Our results demonstrate that it additionally has an important role in regulating the DDC. Interestingly, DDC proteins are known to regulate morphogenetic events during replication stress (Enserink et al., 2006). Our findings therefore highlight a regulatory network where Cla4, which itself seems to be a checkpoint target (Zhou et al., 2016), plays a pivotal role by coordinating cell morphogenesis and reversal of the DDC.

Because reversal of the DDC is crucial for cell survival following DNA damage, similar mechanisms are expected to operate in higher eukaryotes as well. In this regard, different large-scale mass spectrometry studies identified H4T80ph in mammalian cells (Bennetzen et al., 2010; Hornbeck et al., 2015; Lundby et al., 2012; Mertins et al., 2014; Olsen et al., 2010; Parker et al.,

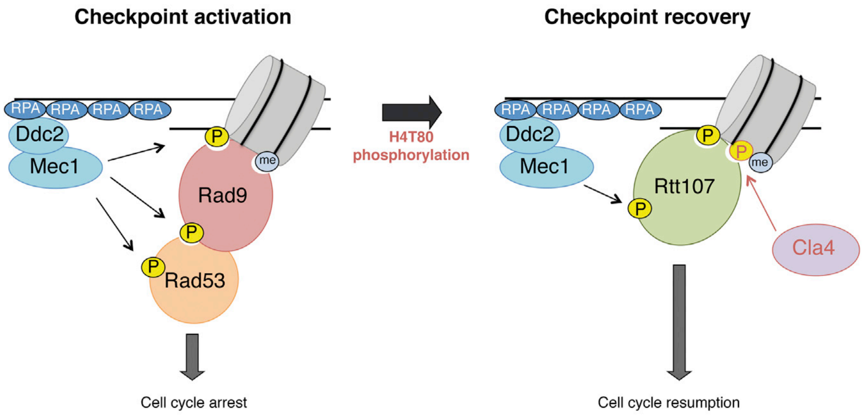

Figure 7. Model of DNA Damage Checkpoint Recovery Initiation

Schematic model depicting the role of H4T80ph in the transition from checkpoint activation to checkpoint recovery. In response to genotoxic stress, Rad9 recruitment to sites of DNA damage, via interaction with H3K79me and γH2A, drives DDC activation. Cla4-dependent H4T80 phosphorylation triggers DDC recovery by recruiting Rtt107, which displaces Rad9 from H3K79me and γH2A, thereby interrupting the signaling cascade.

2015; Tsai et al., 2015). Unfortunately, the specificity of the H4T80ph antibody toward yeast histone H4 sequence did not allow us to extend our studies to human cells (Figure S7C). Nevertheless, there are six PAK family members in humans (PAK1–6; Zhao and Manser, 2012), where PAK1 seems to be the closest homolog of Cla4, because it is known to be involved in cell morphogenesis and cytokinesis and has already been linked to the DDR (Li et al., 2012; Vadlamudi et al., 2004; Yoshizaki et al., 2004). Interestingly, increased PAK1 expression and activity have been well documented in several human cancers (Radu et al., 2014). It is well known that the DDC acts as a barrier during early tumorigenesis, and that its inactivation fosters tumor progression (Bartkova et al., 2005; Gorgoulis et al., 2005). In line with this, PAK1 was shown to promote melanoma chemoresistance by suppressing DNA-damage-sensing pathways (Ho et al., 2012). Further studies will be therefore necessary to determine whether H4T80ph-dependent downregulation of the DDC plays a role in human cancer biology.

STAR★METHODS

Detailed methods are provided in the online version of this paper and include the following:

- KEY RESOURCES TABLE
- CONTACT FOR REAGENT AND RESOURCE SHARING
- EXPERIMENTAL MODEL AND SUBJECT DETAILS
- METHOD DETAILS
  - DNA damage sensitivity assay
  - H4T80ph antibody purification
  - Yeast histone purification
  - Western blot
  - Chromatin Immunoprecipitation (ChIP)
  - Recombinant protein purification
  - *In vitro* phosphorylation assays
  - Flow cytometry analysis
  - RNA library preparation
  - Global differential expression analysis
  - RT-qPCR
  - *In vitro* peptide pull-downs
- QUANTIFICATION AND STATISTICAL ANALYSIS
- DATA AND SOFTWARE AVAILABILITY

SUPPLEMENTAL INFORMATION

Supplemental Information includes seven figures and one table and can be found with this article online at https://doi.org/10.1016/j.molcel.2018.09.023.

ACKNOWLEDGMENTS

We thank M.S. Kobor, M.B. Smolka, and J. Thorner for providing strains and plasmids; A.J. Bannister and J.V. Forment for critically reading the manuscript; J. Jaffe, M.L. Nielsen, and P. Beli for mass spectrometry analysis; and members of Kouzarides and Jackson laboratories for helpful discussions. The Kouzarides laboratory is supported by Cancer Research UK (grant reference RG72100). The Jackson laboratory is supported by Cancer Research UK (grant reference C6/A18796) and Wellcome Trust (Investigator Award 206388/Z/17/Z). Both laboratories benefit from core support from the Wellcome Trust (Core Grant reference WT203144) and Cancer Research UK (grant reference C6946/A24843). G.M.-Z. was funded by an EMBO long-term fellowship (ALTF907-2014).

AUTHOR CONTRIBUTIONS

Conceptualization, G.M.-Z., H.S.-R., and F.P.; Methodology, G.M.-Z., H.S.-R., and F.P.; Investigation, G.M.-Z., H.S.-R., and F.P.; Software, S.C.R.; Formal Analysis, S.C.R.; Writing – Review & Editing, G.M.-Z., H.S.-R., and F.P.; Supervision, S.P.J. and T.K.; Funding Acquisition, S.P.J. and T.K.

DECLARATION OF INTERESTS

T.K. is a co-founder and shareholder of Abcam Plc and co-founder of Storm Therapeutics Ltd., Cambridge, UK.

Received: March 8, 2018
Revised: August 8, 2018
Accepted: September 18, 2018
Published: October 25, 2018

REFERENCES

Andrews, S. (2010). FastQC: a quality control tool for high throughput sequence data (Babraham Bioinformatics). http://www.bioinformatics.babraham.ac.uk/projects/fastqc.

Balint, A., Kim, T., Gallo, D., Cussiol, J.R., Bastos de Oliveira, F.M., Yimit, A., Ou, J., Nakato, R., Gurevich, A., Shirahige, K., et al. (2015). Assembly of Slx4 signaling complexes behind DNA replication forks. EMBO J. *34*, 2182–2197.

Bandyopadhyay, S., Mehta, M., Kuo, D., Sung, M.K., Chuang, R., Jaehnig, E.J., Bodenmiller, B., Licon, K., Copeland, W., Shales, M., et al. (2010).

Rewiring of genetic networks in response to DNA damage. Science *330*, 1385–1389.

Bannister, A.J., and Kouzarides, T. (2011). Regulation of chromatin by histone modifications. Cell Res. *21*, 381–395.

Bartkova, J., Horejsí, Z., Koed, K., Krämer, A., Tort, F., Zieger, K., Guldberg, P., Sehested, M., Nesland, J.M., Lukas, C., et al. (2005). DNA damage response as a candidate anti-cancer barrier in early human tumorigenesis. Nature *434*, 864–870.

Bennett, G., Papamichos-Chronakis, M., and Peterson, C.L. (2013). DNA repair choice defines a common pathway for recruitment of chromatin regulators. Nat. Commun. *4*, 2084.

Bennetzen, M.V., Larsen, D.H., Bunkenborg, J., Bartek, J., Lukas, J., and Andersen, J.S. (2010). Site-specific phosphorylation dynamics of the nuclear proteome during the DNA damage response. Mol. Cell. Proteomics *9*, 1314–1323.

Branzei, D., and Foiani, M. (2006). The Rad53 signal transduction pathway: replication fork stabilization, DNA repair, and adaptation. Exp. Cell Res. *312*, 2654–2659.

Branzei, D., and Foiani, M. (2009). The checkpoint response to replication stress. DNA Repair (Amst.) *8*, 1038–1046.

Chen, C.C., Carson, J.J., Feser, J., Tamburini, B., Zabaronick, S., Linger, J., and Tyler, J.K. (2008). Acetylated lysine 56 on histone H3 drives chromatin assembly after repair and signals for the completion of repair. Cell *134*, 231–243.

Chen, X., Cui, D., Papusha, A., Zhang, X., Chu, C.D., Tang, J., Chen, K., Pan, X., and Ira, G. (2012). The Fun30 nucleosome remodeller promotes resection of DNA double-strand break ends. Nature *489*, 576–580.

Clerici, M., Paciotti, V., Baldo, V., Romano, M., Lucchini, G., and Longhese, M.P. (2001). Hyperactivation of the yeast DNA damage checkpoint by TEL1 and DDC2 overexpression. EMBO J. *20*, 6485–6498.

Cussiol, J.R., Jablonowski, C.M., Yimit, A., Brown, G.W., and Smolka, M.B. (2015). Dampening DNA damage checkpoint signalling via coordinated BRCT domain interactions. EMBO J. *34*, 1704–1717.

Cvrcková, F., De Virgilio, C., Manser, E., Pringle, J.R., and Nasmyth, K. (1995). Ste20-like protein kinases are required for normal localization of cell growth and for cytokinesis in budding yeast. Genes Dev. *9*, 1817–1830.

Dobin, A., Davis, C.A., Schlesinger, F., Drenkow, J., Zaleski, C., Jha, S., Batut, P., Chaisson, M., and Gingeras, T.R. (2013). STAR: ultrafast universal RNA-seq aligner. Bioinformatics *29*, 15–21.

Downs, J.A., Lowndes, N.F., and Jackson, S.P. (2000). A role for Saccharomyces cerevisiae histone H2A in DNA repair. Nature *408*, 1001–1004.

Elledge, S.J. (1996). Cell cycle checkpoints: preventing an identity crisis. Science *274*, 1664–1672.

Enserink, J.M., Smolka, M.B., Zhou, H., and Kolodner, R.D. (2006). Checkpoint proteins control morphogenetic events during DNA replication stress in Saccharomyces cerevisiae. J. Cell Biol. *175*, 729–741.

Giannattasio, M., Lazzaro, F., Plevani, P., and Muzi-Falconi, M. (2005). The DNA damage checkpoint response requires histone H2B ubiquitination by Rad6-Bre1 and H3 methylation by Dot1. J. Biol. Chem. *280*, 9879–9886.

Gilbert, C.S., Green, C.M., and Lowndes, N.F. (2001). Budding yeast Rad9 is an ATP-dependent Rad53 activating machine. Mol. Cell *8*, 129–136.

Gorgoulis, V.G., Vassiliou, L.V., Karakaidos, P., Zacharatos, P., Kotsinas, A., Liloglou, T., Venere, M., Ditullio, R.A., Jr., Kastrinakis, N.G., Levy, B., et al. (2005). Activation of the DNA damage checkpoint and genomic instability in human precancerous lesions. Nature *434*, 907–913.

Hammet, A., Magill, C., Heierhorst, J., and Jackson, S.P. (2007). Rad9 BRCT domain interaction with phosphorylated H2AX regulates the G1 checkpoint in budding yeast. EMBO Rep. *8*, 851–857.

Hammond, S.L., Byrum, S.D., Namjoshi, S., Graves, H.K., Dennehey, B.K., Tackett, A.J., and Tyler, J.K. (2014). Mitotic phosphorylation of histone H3 threonine 80. Cell Cycle *13*, 440–452.

Harper, J.W., and Elledge, S.J. (2007). The DNA damage response: ten years after. Mol. Cell *28*, 739–745.

Ho, H., Aruri, J., Kapadia, R., Mehr, H., White, M.A., and Ganesan, A.K. (2012). RhoJ regulates melanoma chemoresistance by suppressing pathways that sense DNA damage. Cancer Res. *72*, 5516–5528.

Hornbeck, P.V., Zhang, B., Murray, B., Kornhauser, J.M., Latham, V., and Skrzypek, E. (2015). PhosphoSitePlus, 2014: mutations, PTMs and recalibrations. Nucleic Acids Res. *43*, D512–D520.

Hsu, J.Y., Sun, Z.W., Li, X., Reuben, M., Tatchell, K., Bishop, D.K., Grushcow, J.M., Brame, C.J., Caldwell, J.A., Hunt, D.F., et al. (2000). Mitotic phosphorylation of histone H3 is governed by Ipl1/aurora kinase and Glc7/PP1 phosphatase in budding yeast and nematodes. Cell *102*, 279–291.

Jablonowski, C.M., Cussiol, J.R., Oberly, S., Yimit, A., Balint, A., Kim, T., Zhang, Z., Brown, G.W., and Smolka, M.B. (2015). Termination of replication stress signaling via concerted action of the Slx4 scaffold and the PP4 phosphatase. Genetics *201*, 937–949.

Janke, C., Magiera, M.M., Rathfelder, N., Taxis, C., Reber, S., Maekawa, H., Moreno-Borchart, A., Doenges, G., Schwob, E., Schiebel, E., and Knop, M. (2004). A versatile toolbox for PCR-based tagging of yeast genes: new fluorescent proteins, more markers and promoter substitution cassettes. Yeast *21*, 947–962.

Keogh, M.C., Kim, J.A., Downey, M., Fillingham, J., Chowdhury, D., Harrison, J.C., Onishi, M., Datta, N., Galicia, S., Emili, A., et al. (2006). A phosphatase complex that dephosphorylates gammaH2AX regulates DNA damage checkpoint recovery. Nature *439*, 497–501.

Krueger, F. (2012). Trim Galore!: a wrapper tool around Cutadapt and FastQC to consistently apply quality and adapter trimming to FastQ files, with some extra functionality for MspI-digested RRBS-type (reduced representation bisufite-seq) libraries. Babraham Bioinformatics, https://www.bioinformatics.babraham.ac.uk/projects/trim_galore/.

Lawrence, M., Huber, W., Pagès, H., Aboyoun, P., Carlson, M., Gentleman, R., Morgan, M.T., and Carey, V.J. (2013). Software for computing and annotating genomic ranges. PLoS Comput. Biol. *9*, e1003118.

Lawrence, M., Daujat, S., and Schneider, R. (2016). Lateral thinking: how histone modifications regulate gene expression. Trends Genet. *32*, 42–56.

Leberer, E., Dignard, D., Harcus, D., Thomas, D.Y., and Whiteway, M. (1992). The protein kinase homologue Ste20p is required to link the yeast pheromone response G-protein beta gamma subunits to downstream signalling components. EMBO J. *11*, 4815–4824.

Lee, S.E., Moore, J.K., Holmes, A., Umezu, K., Kolodner, R.D., and Haber, J.E. (1998). Saccharomyces Ku70, mre11/rad50 and RPA proteins regulate adaptation to G2/M arrest after DNA damage. Cell *94*, 399–409.

Lee, C.S., Lee, K., Legube, G., and Haber, J.E. (2014). Dynamics of yeast histone H2A and H2B phosphorylation in response to a double-strand break. Nat. Struct. Mol. Biol. *21*, 103–109.

Leroy, C., Lee, S.E., Vaze, M.B., Ochsenbein, F., Guerois, R., Haber, J.E., and Marsolier-Kergoat, M.C. (2003). PP2C phosphatases Ptc2 and Ptc3 are required for DNA checkpoint inactivation after a double-strand break. Mol. Cell *11*, 827–835.

Leung, C.C., and Glover, J.N. (2011). BRCT domains: easy as one, two, three. Cell Cycle *10*, 2461–2470.

Leung, G.P., Brown, J.A., Glover, J.N., and Kobor, M.S. (2016). Rtt107 BRCT domains act as a targeting module in the DNA damage response. DNA Repair (Amst.) *37*, 22–32.

Li, D.Q., Nair, S.S., Ohshiro, K., Kumar, A., Nair, V.S., Pakala, S.B., Reddy, S.D., Gajula, R.P., Eswaran, J., Aravind, L., and Kumar, R. (2012). MORC2 signaling integrates phosphorylation-dependent, ATPase-coupled chromatin remodeling during the DNA damage response. Cell Rep. *2*, 1657–1669.

Love, M.I., Huber, W., and Anders, S. (2014). Moderated estimation of fold change and dispersion for RNA-seq data with DESeq2. Genome Biol. *15*, 550.

Luger, K., Mäder, A.W., Richmond, R.K., Sargent, D.F., and Richmond, T.J. (1997). Crystal structure of the nucleosome core particle at 2.8 A resolution. Nature *389*, 251–260.

Lundby, A., Secher, A., Lage, K., Nordsborg, N.B., Dmytriyev, A., Lundby, C., and Olsen, J.V. (2012). Quantitative maps of protein phosphorylation sites across 14 different rat organs and tissues. Nat. Commun. *3*, 876.

Martín, H., Mendoza, A., Rodríguez-Pachón, J.M., Molina, M., and Nombela, C. (1997). Characterization of SKM1, a Saccharomyces cerevisiae gene encoding a novel Ste20/PAK-like protein kinase. Mol. Microbiol. *23*, 431–444.

Mertins, P., Yang, F., Liu, T., Mani, D.R., Petyuk, V.A., Gillette, M.A., Clauser, K.R., Qiao, J.W., Gritsenko, M.A., Moore, R.J., et al. (2014). Ischemia in tumors induces early and sustained phosphorylation changes in stress kinase pathways but does not affect global protein levels. Mol. Cell. Proteomics *13*, 1690–1704.

Mok, J., Kim, P.M., Lam, H.Y., Piccirillo, S., Zhou, X., Jeschke, G.R., Sheridan, D.L., Parker, S.A., Desai, V., Jwa, M., et al. (2010). Deciphering protein kinase specificity through large-scale analysis of yeast phosphorylation site motifs. Sci. Signal. *3*, ra12.

Muzi Falconi, M., Piseri, A., Ferrari, M., Lucchini, G., Plevani, P., and Foiani, M. (1993). De novo synthesis of budding yeast DNA polymerase alpha and POL1 transcription at the G1/S boundary are not required for entrance into S phase. Proc. Natl. Acad. Sci. USA *90*, 10519–10523.

Nakada, D., Hirano, Y., Tanaka, Y., and Sugimoto, K. (2005). Role of the C terminus of Mec1 checkpoint kinase in its localization to sites of DNA damage. Mol. Biol. Cell *16*, 5227–5235.

Navadgi-Patil, V.M., and Burgers, P.M. (2009). The unstructured C-terminal tail of the 9-1-1 clamp subunit Ddc1 activates Mec1/ATR via two distinct mechanisms. Mol. Cell *36*, 743–753.

O'Neill, B.M., Szyjka, S.J., Lis, E.T., Bailey, A.O., Yates, J.R., 3rd, Aparicio, O.M., and Romesberg, F.E. (2007). Pph3-Psy2 is a phosphatase complex required for Rad53 dephosphorylation and replication fork restart during recovery from DNA damage. Proc. Natl. Acad. Sci. USA *104*, 9290–9295.

Ohouo, P.Y., Bastos de Oliveira, F.M., Liu, Y., Ma, C.J., and Smolka, M.B. (2013). DNA-repair scaffolds dampen checkpoint signalling by counteracting the adaptor Rad9. Nature *493*, 120–124.

Olsen, J.V., Vermeulen, M., Santamaria, A., Kumar, C., Miller, M.L., Jensen, L.J., Gnad, F., Cox, J., Jensen, T.S., Nigg, E.A., et al. (2010). Quantitative phosphoproteomics reveals widespread full phosphorylation site occupancy during mitosis. Sci. Signal. *3*, ra3.

Parker, B.L., Yang, G., Humphrey, S.J., Chaudhuri, R., Ma, X., Peterman, S., and James, D.E. (2015). Targeted phosphoproteomics of insulin signaling using data-independent acquisition mass spectrometry. Sci. Signal. *8*, rs6.

Pellicioli, A., and Foiani, M. (2005). Signal transduction: how rad53 kinase is activated. Curr. Biol. *15*, R769–R771.

Pfander, B., and Diffley, J.F. (2011). Dpb11 coordinates Mec1 kinase activation with cell cycle-regulated Rad9 recruitment. EMBO J. *30*, 4897–4907.

Puddu, F., Granata, M., Di Nola, L., Balestrini, A., Piergiovanni, G., Lazzaro, F., Giannattasio, M., Plevani, P., and Muzi-Falconi, M. (2008). Phosphorylation of the budding yeast 9-1-1 complex is required for Dpb11 function in the full activation of the UV-induced DNA damage checkpoint. Mol. Cell. Biol. *28*, 4782–4793.

Puddu, F., Piergiovanni, G., Plevani, P., and Muzi-Falconi, M. (2011). Sensing of replication stress and Mec1 activation act through two independent pathways involving the 9-1-1 complex and DNA polymerase ε. PLoS Genet. *7*, e1002022.

Radu, M., Semenova, G., Kosoff, R., and Chernoff, J. (2014). PAK signalling during the development and progression of cancer. Nat. Rev. Cancer *14*, 13–25.

Shimada, K., Oma, Y., Schleker, T., Kugou, K., Ohta, K., Harata, M., and Gasser, S.M. (2008). Ino80 chromatin remodeling complex promotes recovery of stalled replication forks. Curr. Biol. *18*, 566–575.

St Onge, R.P., Mani, R., Oh, J., Proctor, M., Fung, E., Davis, R.W., Nislow, C., Roth, F.P., and Giaever, G. (2007). Systematic pathway analysis using high-resolution fitness profiling of combinatorial gene deletions. Nat. Genet. *39*, 199–206.

Sugawara, N., Wang, X., and Haber, J.E. (2003). In vivo roles of Rad52, Rad54, and Rad55 proteins in Rad51-mediated recombination. Mol. Cell *12*, 209–219.

Symington, L.S. (2002). Role of RAD52 epistasis group genes in homologous recombination and double-strand break repair. Microbiol. Mol. Biol. Rev. *66*, 630–670.

Tessarz, P., Santos-Rosa, H., Robson, S.C., Sylvestersen, K.B., Nelson, C.J., Nielsen, M.L., and Kouzarides, T. (2014). Glutamine methylation in histone H2A is an RNA-polymerase-I-dedicated modification. Nature *505*, 564–568.

Tsai, C.F., Wang, Y.T., Yen, H.Y., Tsou, C.C., Ku, W.C., Lin, P.Y., Chen, H.Y., Nesvizhskii, A.I., Ishihama, Y., and Chen, Y.J. (2015). Large-scale determination of absolute phosphorylation stoichiometries in human cells by motif-targeting quantitative proteomics. Nat. Commun. *6*, 6622.

Vadlamudi, R.K., Li, F., Barnes, C.J., Bagheri-Yarmand, R., and Kumar, R. (2004). p41-Arc subunit of human Arp2/3 complex is a p21-activated kinase-1-interacting substrate. EMBO Rep. *5*, 154–160.

van Leeuwen, F., Gafken, P.R., and Gottschling, D.E. (2002). Dot1p modulates silencing in yeast by methylation of the nucleosome core. Cell *109*, 745–756.

Vaze, M.B., Pellicioli, A., Lee, S.E., Ira, G., Liberi, G., Arbel-Eden, A., Foiani, M., and Haber, J.E. (2002). Recovery from checkpoint-mediated arrest after repair of a double-strand break requires Srs2 helicase. Mol. Cell *10*, 373–385.

Versele, M., and Thorner, J. (2004). Septin collar formation in budding yeast requires GTP binding and direct phosphorylation by the PAK, Cla4. J. Cell Biol. *164*, 701–715.

Wang, X., and Haber, J.E. (2004). Role of Saccharomyces single-stranded DNA-binding protein RPA in the strand invasion step of double-strand break repair. PLoS Biol. *2*, E21.

Yoshizaki, H., Ohba, Y., Parrini, M.C., Dulyaninova, N.G., Bresnick, A.R., Mochizuki, N., and Matsuda, M. (2004). Cell type-specific regulation of RhoA activity during cytokinesis. J. Biol. Chem. *279*, 44756–44762.

Yu, G., Wang, L.G., Han, Y., and He, Q.Y. (2012). clusterProfiler: an R package for comparing biological themes among gene clusters. OMICS *16*, 284–287.

Zhao, Z.S., and Manser, E. (2012). PAK family kinases: physiological roles and regulation. Cell. Logist. *2*, 59–68.

Zhou, C., Elia, A.E., Naylor, M.L., Dephoure, N., Ballif, B.A., Goel, G., Xu, Q., Ng, A., Chou, D.M., Xavier, R.J., et al. (2016). Profiling DNA damage-induced phosphorylation in budding yeast reveals diverse signaling networks. Proc. Natl. Acad. Sci. USA *113*, E3667–E3675.

Zou, L., and Elledge, S.J. (2003). Sensing DNA damage through ATRIP recognition of RPA-ssDNA complexes. Science *300*, 1542–1548.

STAR★METHODS

KEY RESOURCES TABLE

REAGENT or RESOURCE	SOURCE	IDENTIFIER
Antibodies		
Anti-H4T80ph	Orygen	This study; RRID: AB_2313773
Anti-H4	Abcam	RRID:AB_296888
Anti-Rad53	Abcam	RRID:AB_2687603
Anti-γH2A	Abcam	RRID:AB_301630
Anti-HA	Abcam	RRID:AB_307019
Anti-FLAG	Sigma	RRID:AB_262044
Bacterial and Virus Strains		
BL21 (DE3)	New England Biolabs	Cat. No. C2527I
Chemicals, Peptides, and Recombinant Proteins		
H4T80 peptide	GeneCust	This study
H4T80ph peptide	GeneCust	This study
H4T30ph peptide	GeneCust	This study
H4T96ph peptide	GeneCust	This study
Biotinylated H4T80 peptide	GeneCust	This study
Biotinylated H4T80ph peptide	GeneCust	This study
Human H4T80 peptide	GeneCust	This study
Human H4T80ph peptide	GeneCust	This study
Sulfolink Coupling Resin	ThermoFisher	Cat.No. 20402
Protease inhibitors	Roche	Cat.No. 11697498001
Phosphatase inhibitors	Roche	Cat.No. 04906845001
Camptothecin	Sigma	Cat.No.C9911
Methyl methanesulfonate	Sigma	Cat.No.129925
α-factor	GenScript	Cat.No.RP01002
Sytox Green	Invitrogen	Cat.No.S7020
Dynabeads MyOne Streptavidin C1	ThermoFisher	Cat.No. 65001
Calf histones	Roche	Cat.No. 10223565001
DNase I	Zymo Research	Cat.No E1009-A
NEXTflex Poly(A) beads	Bioo Scientific	NOVA-512981
Critical Commercial Assays		
Fast SYBR Green Master Mix	ThermoFisher	Cat.No.4385612
NEXTflex RNA-seq kit	Bioo Scientific	NOVA-512913
Deposited Data		
RNA-seq data	This study	https://www.ebi.ac.uk/arrayexpress/experiments/E-MTAB-7090/
Unprocessed imaging data	This study	https://doi.org/10.17632/yjwrsb92fg.1
Experimental Models: Organisms/Strains		
S. cerevisiae wild type and mutant strains background: W303	This study	Table S1
Oligonucleotides		
qPCR 0.2 kb from HO<br>up: CCCATCGTCTTGCTCTTGTT<br>low: ATCCGTCCCGTATAGCCAAT	This study	N/A
qPCR 1 kb from HO up: CAAGGATGCCCTTGTTTTGT low: TTTTGACGGCCAATCTTTTC	This study	N/A

(*Continued on next page*)

Continued		
REAGENT or RESOURCE	SOURCE	IDENTIFIER
qPCR 5 kb from HO	This study	N/A
up: CCAAGGAACTAATGATCTAAGCACA		
low: CATGTTGGTACTCTAAATCACCTCC		
qPCR *PRP8*	This study	N/A
up: GAGTGTGGCTAAATTTCTTAAGAGG		
low: TCGAATACTCTCAGGCATCATTTCT		
qPCR *RTT107*	This study	N/A
up: CTTGCTACTTTGTGTGAGCTTGAT		
low: TTTGTTTGGATGAAGAGTAAGCTG		
qPCR *RNR3*	This study	N/A
up: AGGTCGTGGTAAAACAATTAAAGC		
low: TGTTGGTTTGTCTTCCTGTTACAT		
qPCR *ACT1*	This study	N/A
up: GAAATGCAAACCGCTGCTCA		
low: TACCGGCAGATTCCAAACCC		
Recombinant DNA		
pMR206 (*TRP1-HHT2-HHF2)*	(Tessarz et al., 2014)	N/A
pMBV80 (GST-*CLA4)*	(Versele and Thorner, 2004)	N/A
pMBV81 (GST-*CLA4K594A)*	(Versele and Thorner, 2004)	N/A
pMBS362 (*RAD53-R605A::kanMX6)*	(Ohouo et al., 2013)	N/A
Software and Algorithms		
fastQC	(Andrews, 2010)	N/A
trim-galore	(Krueger, 2012)	N/A
STAR universal RNA seq aligner	(Dobin et al., 2013)	N/A
GenomicAlignments package	(Lawrence et al., 2013)	N/A
DESeq2 package	(Love et al., 2014)	N/A
clusterProfiler package	(Yu et al., 2012)	N/A

CONTACT FOR REAGENT AND RESOURCE SHARING

Further information and requests for resources and reagents should be directed to and will be fulfilled by the Lead Contact, Prof. Tony Kouzarides (t.kouzarides@gurdon.cam.ac.uk).

EXPERIMENTAL MODEL AND SUBJECT DETAILS

All *Saccharmoyces cerevisae* strains used in this study are derivatives of W303 background. Integrations and deletions were performed using one-step PCR-based methods (Janke et al., 2004). Histone point mutants were shuffled by counter-selection on 5-FOA. Genotypes are listed in Table S1. All strains were routinely grown in YPAD at 30°C.

METHOD DETAILS

DNA damage sensitivity assay

Yeast strains were cultured overnight to stationary phase. Cultures were then diluted to OD_{600} = 0.5 in sterile water. 10-fold serial dilutions were prepared and 5 μl volumes were spotted onto the corresponding plates. Images were taken 2-3 days later.

H4T80ph antibody purification

The H4T80ph antibody was produced using a 90-day sheep immunization protocol (Orygen Antibodies Ltd, UK). Unmodified and modified peptides columns were prepared by coupling either H4T80 or H4T80ph peptides to a sulfolink resin. Sera were first incubated with H4T80 column for 1 hour on rotation at room temperature. Supernatant was recovered and incubated with H4T80ph column for 1 hour on rotation at room temperature. H4T80ph column was then washed 6 times with TBS, and antibodies were eluted

in 100mM Glycine pH 2.5. Elution was quickly neutralized with ice-cold 1M TrisHCl ph 8.8 solution, and dialyzed overnight in TBS + 10% glycerol.

Yeast histone purification

Yeast strains were cultured overnight to stationary phase. Next day, cells were grown in 1l of YPD for 3-4 generations to OD_{600} = 1. Cells were then collected, washed with water and frozen in liquid nitrogen. The cell pellet was resuspended in SP buffer (1M Sorbitol; 50mM potassium phosphate pH 6.8, 14mM β-mercaptoethanol) and spheroplasted by zymolyase digestion. Nuclei were then prepared by douncing in Lysis buffer (18% Ficoll-400 [w/v]; 20mM potassium phosphate pH 6.8; 1mM $MgCl_2$; 0.5mM EDTA) supplemented with both protease and phosphatase inhibitors (Roche). After spinning down in a benchtop centrifuge, supernatant was recovered and nuclei were pelleted by spinning at 20,000 rpm for 30 minutes in a Beckman SW-41 Ti rotor. Nuclei were then resuspended in NP buffer (0.34M sucrose; 20mM Tris-HCl pH 7.4; 50mM KCl; 5mM $MgCl_2$) supplemented with both protease and phosphatase inhibitors (Roche) and pelleted by spinning at 17,000 rpm for 30 minutes in a Beckman SW-41 Ti rotor. Nuclei were washed three times with Buffer A (10mM Tris-HCl pH 8.0; 0.5% NP-40 [v/v]; 75mM NaCl) supplemented with both protease and phosphatase inhibitors (Roche). Histones were then extracted in Buffer B (10mM Tris-HCl pH 8; 400mM NaCl; 0.2M H_2SO_4) for 1 hour on rotation at 4°C and TCA precipitated.

Western blot

Total protein extracts were prepared as previously described (Muzi Falconi et al., 1993). Cells were washed twice with 1 mL of 20% [w/v] TCA and disrupted by vortexing for 4 minutes using acid-washed glass beads. Extracts were then neutralized by adding 1M Tris base and boiled in Laemmli buffer. For Rad53 and Rtt107 phosphorylation analysis, total protein extracts were separated by sodium dodecyl sulfate-polyacrylamide gel electrophoresis (SDS-PAGE) in 7% acrylamide gels. Rad9 phosphorylation analysis was performed using NuPAGE Tris-acetate 3 to 8% gels and following the manufacturer's instructions (ThermoFisher). For H4T80 phosphorylation analysis, purified yeast histones were separated by SDS-PAGE in 17% acrylamide gels. Every experiment was repeated at least two (three in most cases) independent times. Representative blots are shown.

Chromatin Immunoprecipitation (ChIP)

For the inducible DSB experiments, the corresponding yeast strains were grown in YP medium containing 2% raffinose until they reached OD_{600} = 0.5. Then, 2% galactose was added to induce expression of the HO endonuclease and 100 mL of yeast cultures per ChIP experiment were collected at the indicated times. Cells were cross-linked with 1% formaldehyde for 15 minutes at room temperature, and the reaction was quenched with 125mM glycine. Cells were resuspended in ChIP SDS buffer (1% SDS, 10 mM EDTA, 50 mM Tris HCl pH 8.0) supplemented with protease inhibitors (Roche) and disrupted with glass beads by using a FastPrep instrument (MP Biomedicals). Chromatin was sonicated (Bioruptor Pico, Diagenode; 10 cycles, 30 s on/off) to yield an average DNA fragment of 300-500 base pairs, and diluted 10 times in ChIP IP buffer (0.01% SDS, 1.1%Triton X-100, 167mM NaCl, 1.2mM EDTA, 16.7mM Tris HCl pH 8.0) supplemented with protease inhibitors (Roche) prior to overnight immunoprecipitation on rotation at 4°C. Next day, 50μl of protein G dynabeads were added, and samples were incubated again on rotation at 4°C for 2 hours. Then, dynabeads were washed twice with the following buffers: TSE 150 (1% Triton X-100, 0.1%SDS, 150 mM NaCl 2 mM EDTA, 20 mM Tris HCl pH 8.0), TSE 500 (1% Triton X-100, 0.1%SDS, 500 mM NaCl 2 mM EDTA, 20 mM Tris HCl pH 8.0) and LiCl buffer (0.25 M LiCl, 1% NP-40, 1% deoxycholate, 1 mM EDTA, 10 mM Tris HCl pH 8.0). DNA was eluted at 65°C in elution buffer (100 mM $NaHCO_3$, 1% SDS), and cross-linking was reverted by overnight incubation at 65°C. Samples were treated with 0.5 mg/ml of RNase A at 37°C for 2 h. DNA was purified using the ChIP DNA Clean & Concentrator kit (Zymo Research). Relative DNA amounts were determined by qPCR using Fast SYBR Green Master Mix (Applied Biosystems). Primer pairs used for amplification are listed in the Key Resources Table. For each strain and/or condition, three independent colonies were grown and processed. The mean values +SEM derived from three biological replicates were plotted using Prism (GraphPad Software, Inc.).

Recombinant protein purification

Plasmids are listed in the Key Resources Table. Expression of either GST-Cla4 or GST-Cla4K594A fusions in BL21 (DE3) cells was induced with 0.2mM IPTG for 4h at 20°C. Cells were then lysed by sonication in PBS + 0.1% [v/v] Tween-20 supplemented with protease inhibitors, and clarified lysate was incubated with glutathione-agarose beads for 1h at 4°C. Immobilized GST fusion proteins were washed extensively with PBS + 0.1% [v/v] Tween 20 and then with PBS. Immobilized GST-Cla4 and GST-Cla4K594A were then used for kinase reactions.

In vitro phosphorylation assays

Kinase reactions were performed for 1h at 30°C in Kinase buffer (50mM Tris-HCl pH 7.5; 10mM $MgCl_2$; 1mM DTT; 150mM NaCl; 50mM β-glycerolphosphate; 0.05% [v/v] NP-40; 50mM cold ATP and 0.37MBq (10μCi Υ^{32}P-ATP), using 35 ng of GST-Cla4 or GST-Cla4K594A enzyme and 1μg calf H4, 4μg calf histone mixture (H2A, H2B, H3, H4), 2μg H4 peptide (residues 70-90) or 3μg of H4T80A peptide (residues 70-90) as substrates. Reactions on histones were separated by SDS-PAGE in 17% acrylamide gels, whereas reactions on peptides were resolved in 17% acrylamide Tricine gels. Gels were stained with Coomassie brilliant blue to visualize proteins and peptides, dried and exposed to film. Bands were quantified using ImageJ software. The mean values +SEM derived from two independent experiments were plotted using Prism (GraphPad Software, Inc.).

Flow cytometry analysis

Cell pellets were resuspended in ice-cold 70% [v/v] ethanol and incubated at 4°C overnight. Cells were then washed with 50mM Tris-HCl pH 8.0, and incubated with 0.4mg/ml RNase A at 37°C overnight. After treatment with pepsin for 30 min at 37°C, cells were resuspended in 1μM Sytox Green solution and analyzed using BD FACS Calibur flow cytometer.

RNA library preparation

For each strain, three independent colonies were grown in YPD medium to OD_{600} = 0.6, and total RNA was prepared by hot-phenol extraction. RNA was subjected to DNase I treatment (catalog number E1009-A; Zymo Research) and poly-A mRNA was purified using poly-T oligo-attached magnetic beads (NEXTflex® Poly(A) Beads, catalog number NOVA-512981) according to the manufacturer's instructions. RNA quantity and purity were assessed using an Agilent high-sensitivity RNA screen tape system (catalog number 5067-5579; Agilent Technologies) and Qubit (Molecular Probes, Invitrogen). 200 ng of polyA were used to prepare libraries with the NEXTflex RNA-seq kit (NOVA-512913, Illumina) according to the manufacturer's instructions. Samples were barcoded and combined at uniform molarity to create a single pool, which was sequenced in a single-end 75-bp run on an Illumina NextSeq machine.

Global differential expression analysis

Quality control of raw fastq reads was conducted using fastQC (Andrews, 2010). Raw reads were trimmed to remove adaptor contamination and poor-quality bases using trim-galore (Krueger, 2012) with parameters "–illumina -q 20–stringency 5 -e 0.1–length 30–trim-n." Trimmed reads were mapped against the R64-1-1 *Saccharomyces cerevisiae* genome using the STAR universal RNA seq aligner (Dobin et al., 2013) with parameters "–outSAMmultNmax 300–outSAMstrandField intronMotif." All differential gene expression analyses were conducted in R. Gene counts were generated for all samples at the transcript level by using the summarizeOverlaps function in the GenomicAlignments package (Lawrence et al., 2013) using mode "Union." Gene models for all transcripts were taken from the Ensembl v91 R64-1-1 *Saccharomyces cerevisiae* dataset. Differential gene expression analysis was conducted for H4T80A mutant samples versus wild-type samples using the DESeq2 package (Love et al., 2014). P values were adjusted using the Benjamini and Hochberg multiple testing correction. Significantly differentially expressed genes were identified based on a fold-change of 2-fold or greater (up- or downregulated) and an adjusted p value less than 0.05. Gene ontology analysis was conducted using the clusterProfiler package (Yu et al., 2012).

RT-qPCR

For each strain, three independent colonies were grown in YPD medium to OD_{600} = 0.4. Cells were arrested in G1 using α-factor, and then released in the presence of 20μM CPT for 45 min. Total RNA was prepared by hot-phenol extraction, and RNA quantity and purity were assessed using a NanoDrop 1000 instrument. 10 μg of total RNA were treated with TURBO DNase (Invitrogen; Catalog number AM2238). RNA was purified using RNA Clean & Concentrator Kit (Zymo; Catalog number R1016), and cDNA was prepared using Superscript III reverse transcriptase (Invitrogen; Catalog number 18080). Expression levels of individual transcripts were determined by qPCR using Fast SYBR Green Master Mix (Thermo; Catalog number 4385612) and oligonucleotides listed in the Key Resources Table. Relative levels were determined by normalization to the *ACT1* mRNA in each sample. The mean values +SEM derived from the three biological replicates were plotted using Prism (GraphPad Software, Inc.).

In vitro peptide pull-downs

Yeast cells were grown to exponential phase and whole cell extracts were prepared using glass beads in binding buffer (20mM HEPES pH 7.9; 150mM NaCl; 1% [v/v] NP-40; 1mM DTT; 20% [v/v] glycerol) supplemented with both protease and phosphatase inhibitors. For peptide immunoprecipitation, 4μg of the corresponding peptide were diluted in 1ml of binding buffer and incubated with 75μl of Dynabeads MyOne Streptavidin C1 for 60 minutes at room temperature. Beads were washed twice with binding buffer. 0.5mg of whole cell extract was then incubated with beads for 3 hours at 4°C and washed 6 times with 300mM NaCl binding buffer. Immunoprecipitated proteins were then separated by SDS-PAGE in a 17% acrylamide gel.

QUANTIFICATION AND STATISTICAL ANALYSIS

ImageJ was used for quantification of *in vitro* kinase assays. Microsoft excel software was used to perform all statistical analyses. Statistical differences were determined by two-tailed Student t test. Significance is denoted as * for $p < 0.05$.

DATA AND SOFTWARE AVAILABILITY

The accesion number for the RNA-seq data reported in this paper is [ArrayExpress]: [E-MTAB-7090].

Unprocessed imaging data are deposited on Mendeley Data:
https://doi.org/10.17632/yjwrsb92fg.1

Molecular Cell, Volume *72*

Supplemental Information

Phosphorylation of Histone H4T80 Triggers DNA Damage Checkpoint Recovery

Gonzalo Millan-Zambrano, Helena Santos-Rosa, Fabio Puddu, Samuel C. Robson, Stephen P. Jackson, and Tony Kouzarides

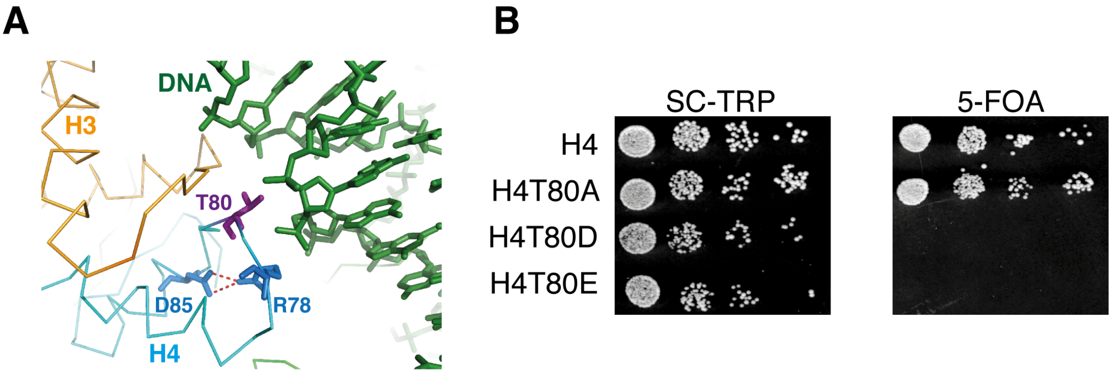

Figure S1. Histone H4T80 phosphorylation levels must be tightly regulated. Related to Figure 1. (A) Ribbon structure of H3 L1 loop and H4 L2 loop indicating hydrogen bonds between H4R78 and H4D85. **(B)** Spot test for cell viability. 10-fold serial dilutions were used.

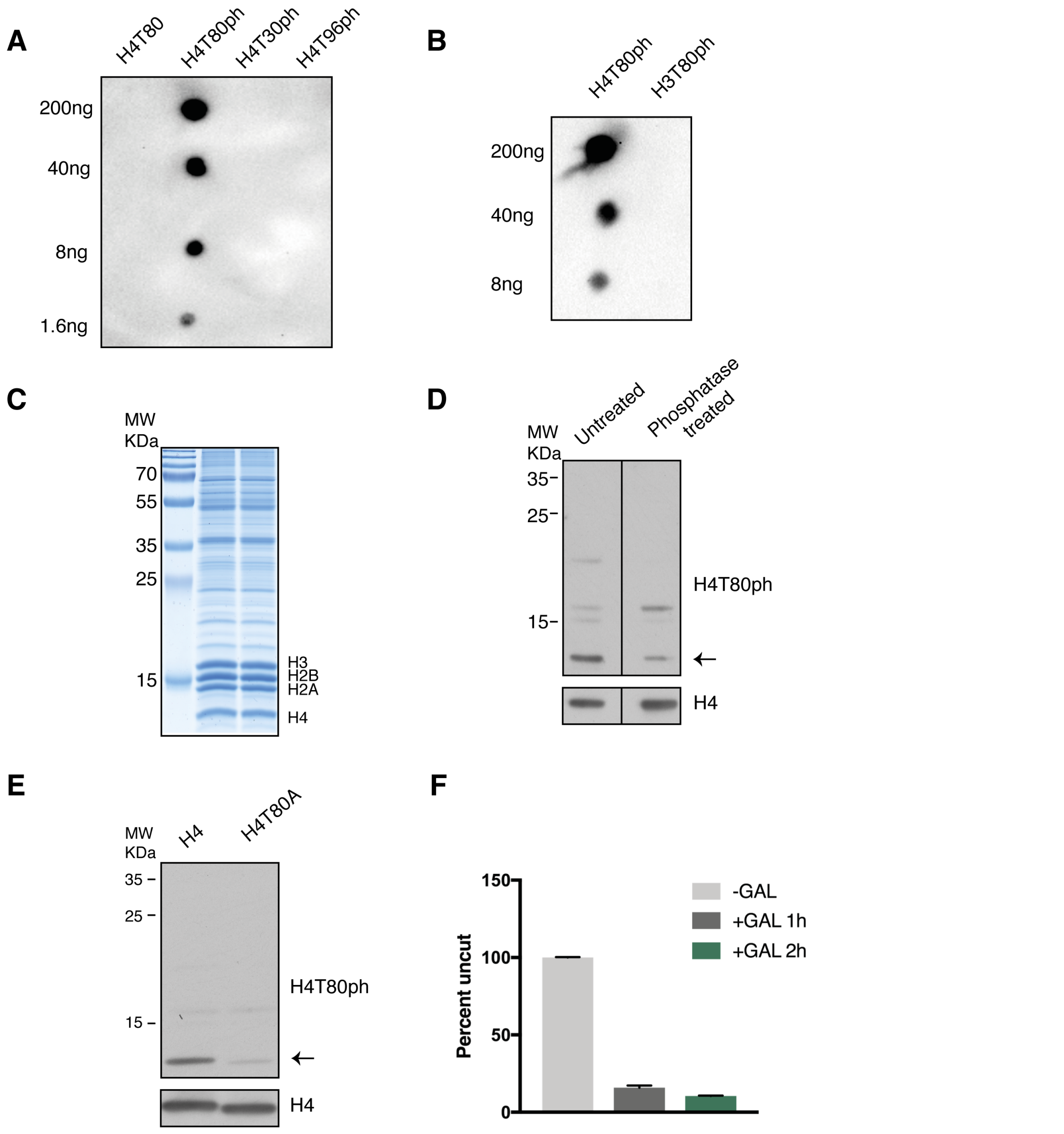

Figure S2. Anti-H4T80ph antibody specifically recognizes H4T80ph in yeast. Related to Figure 1. (A) and **(B)** Dot-blot analysis using H4T80ph specific antibody. 5-fold serial dilutions of different peptides, as indicated, were spotted onto a PVDF membrane. **(C)** Coomassie blue stained gel showing purified yeast histones separated by SDS-PAGE in 17% acrylamide gels. **(D)** Immunoblot analysis of purified yeast histones separated by SDS-PAGE in 17% acrylamide gels. Nitrocellulose membranes were treated or not with alkaline phosphatase. Blots were probed with anti-H4T80ph antibody and then re-probed with anti-H4 antibody as indicated. ← Corresponds to histone H4. **(E)** Immunoblot analysis of purified yeast histones separated by SDS-PAGE in 17% acrylamide gels. Both wild type and H4T80A mutant cells were grown to exponential phase. Blots were probed with anti-H4T80ph antibody and then re-probed with anti-H4 antibody as indicated. **(F)** HO cutting efficiency was measured by qPCR using primers encompassing the HO cut site and normalized to the *PRP8* reference locus. The mean of the non-induced samples was calculated as 100% uncut. Graphs show mean +SEM of three biological replicates.

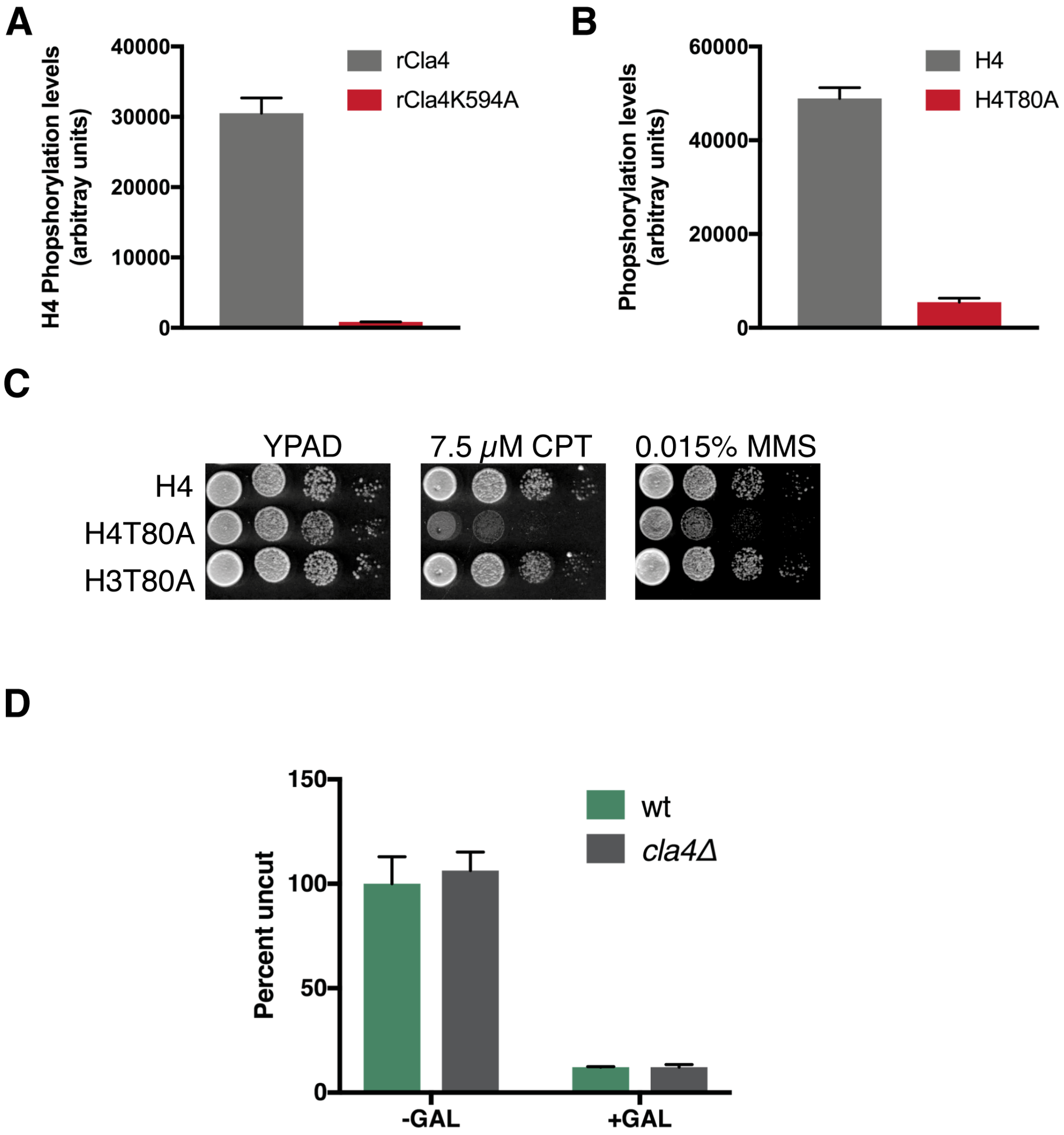

Figure S3. Cla4 phosphorylates H4T80 *in vivo* and *in vitro*. Related to Figure 2 and Figure 3. (A) and **(B)** Quantification of kinase experiments shown in Figure 2D and 2E respectively. Data are represented as mean +SEM of two independent experiments. **(C)** Spot test for DNA damage sensitivity of different yeast histone point mutants as indicated. 10-fold serial dilutions were used. **(D)** HO cutting efficiency was measured by qPCR using primers encompassing the HO cut site and normalized to the *PRP8* reference locus. The mean of the non-induced samples was calculated as 100% uncut. Graphs show mean +SEM of three biological replicates.

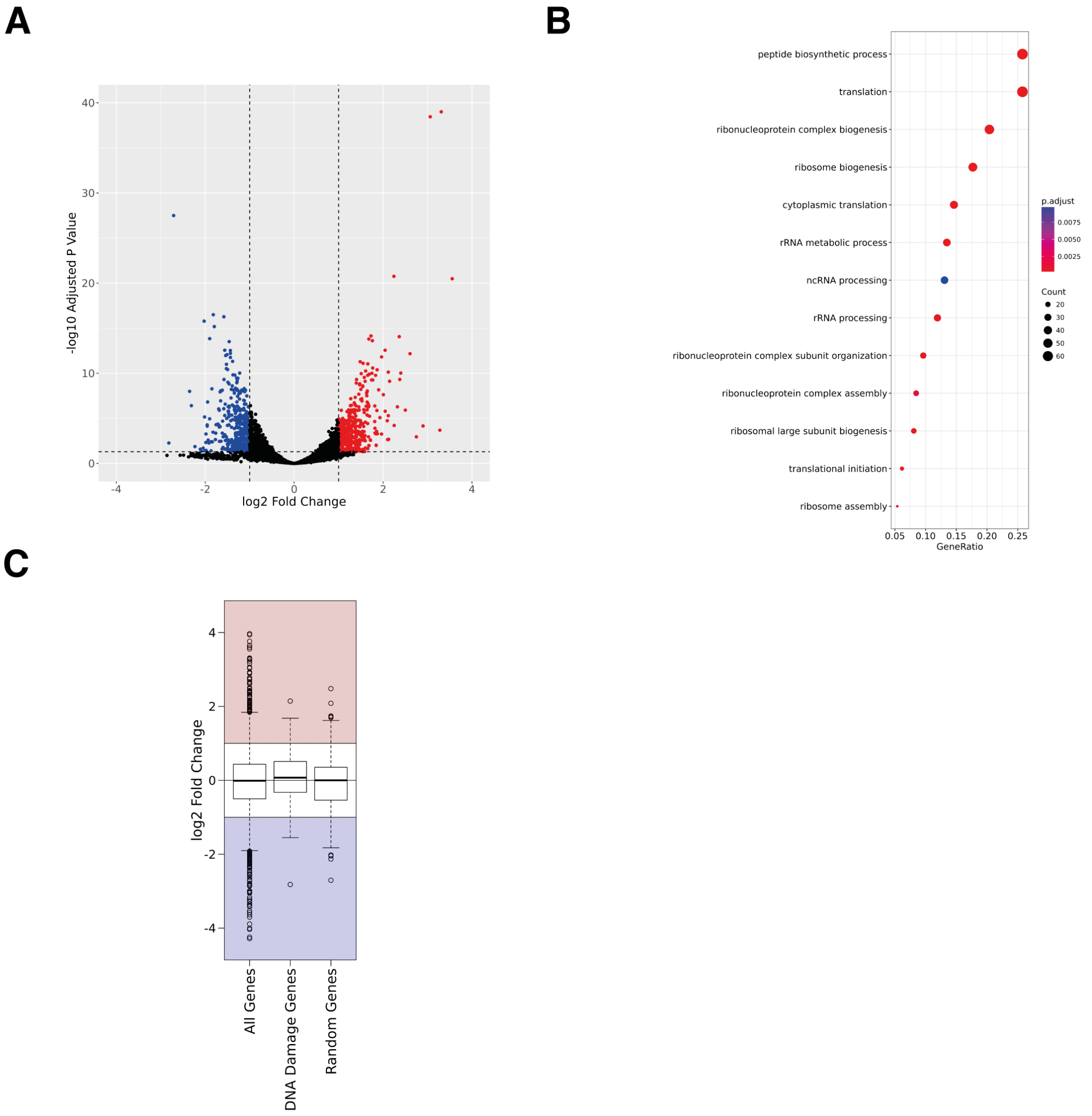

Figure S4. H4T80A mutation does not particularly impair the expression of genes involved in the DDR. Related to Figure 4. (A) Volcano plot showing the global differential expression between H4T80A mutant and wild type samples. The x-axis represents the log2 fold change and the y-axis represents the -log10 of the adjusted p-value from the DESeq2 differential expression analysis. Significantly up-regulated (fold change > 2, adjusted p value < 0.05) and down-regulated (fold change < 2, adjusted p value < 0.05) genes are highlighted in red and blue respectively. **(B)** Dot-plot of gene ontology terms enriched for genes down-regulated in H4T80A mutant cells compared to wild type. For each term, GeneRatio represents the proportion of genes in a given GO term that are significantly down-regulated. The size of the dots is representative of the total number of genes, whilst the colour is representative of the p value from the over-representation test. Genes up-regulated in the H4T80A mutant show no enrichment in the GO analysis. **(C)** Boxplot showing the distribution of log2 fold change values from the DESeq2 differential expression analysis for; all genes, 206 genes involved in the DNA damage response (based on GO term GO:0006974), and a random subset of 206 genes. Genes up- or down-regulated greater than 2-fold are highlighted in red or blue respectively.

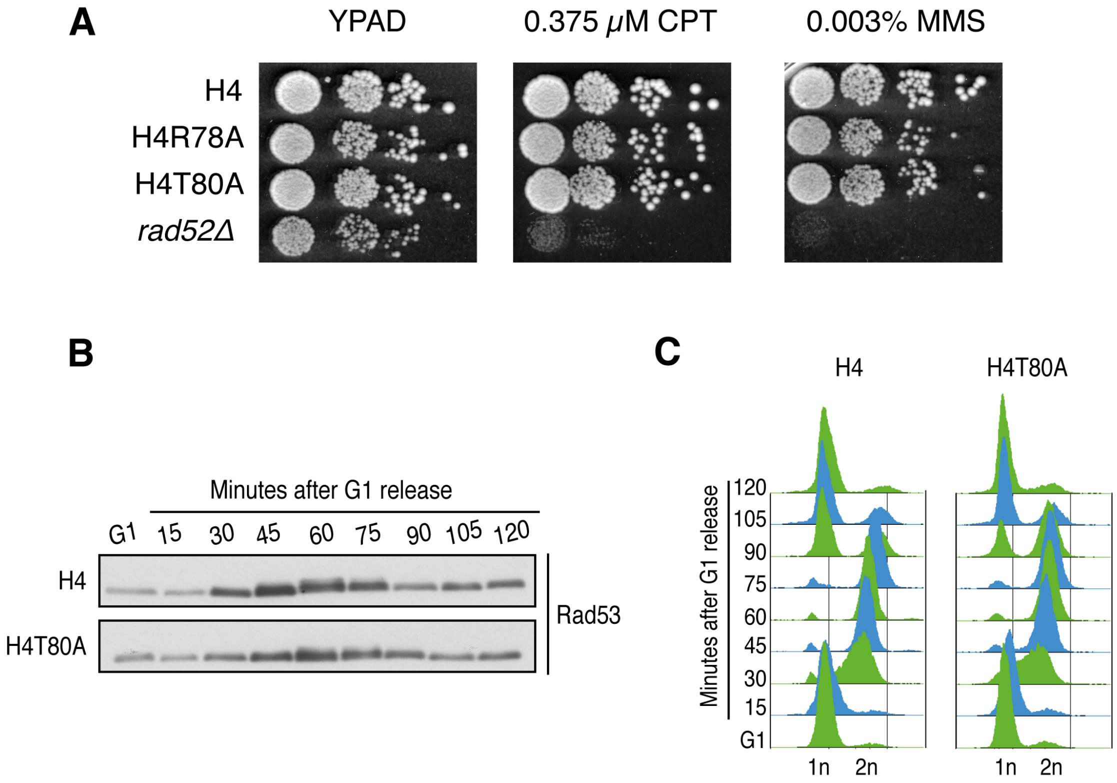

Figure S5. H4T80A mutation does not impair cell cycle progression. Related to Figure 4. (A) Spot test for DNA damage sensitivity of different yeast mutants as indicated. 10-fold serial dilutions were used. **(B)** Immunoblot analysis of Rad53 phosphorylation levels evaluated by mobility shift in SDS-PAGE gels. Wild type or H4T80A mutant cells were arrested in G1 using α-factor, and then released for 120 min. Samples were taken every 15 min. α-factor was added again after 45 min to arrest cells in G1. **(C)** Flow cytometry analysis of the same samples taken in B.

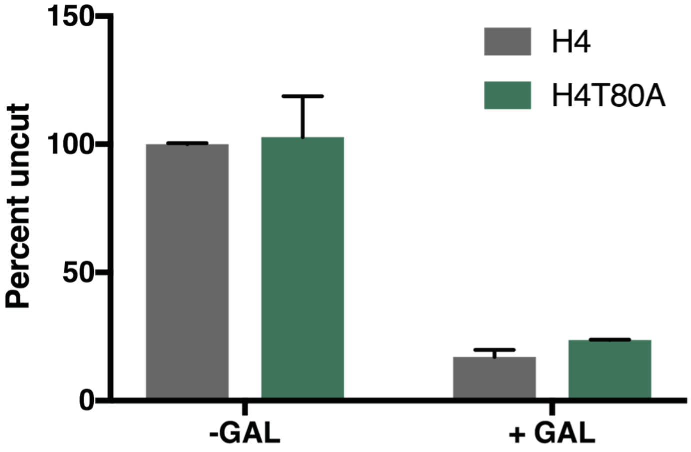

Figure S6. DSB is induced to the same extent in H4T80A mutant and wild type cells. Related to Figure 5. HO cutting efficiency was measured by qPCR using primers encompassing the HO cut site and normalized to the *PRP8* reference locus. The mean of the non-induced samples was calculated as 100% uncut. Graphs show mean +SEM of two biological replicates.

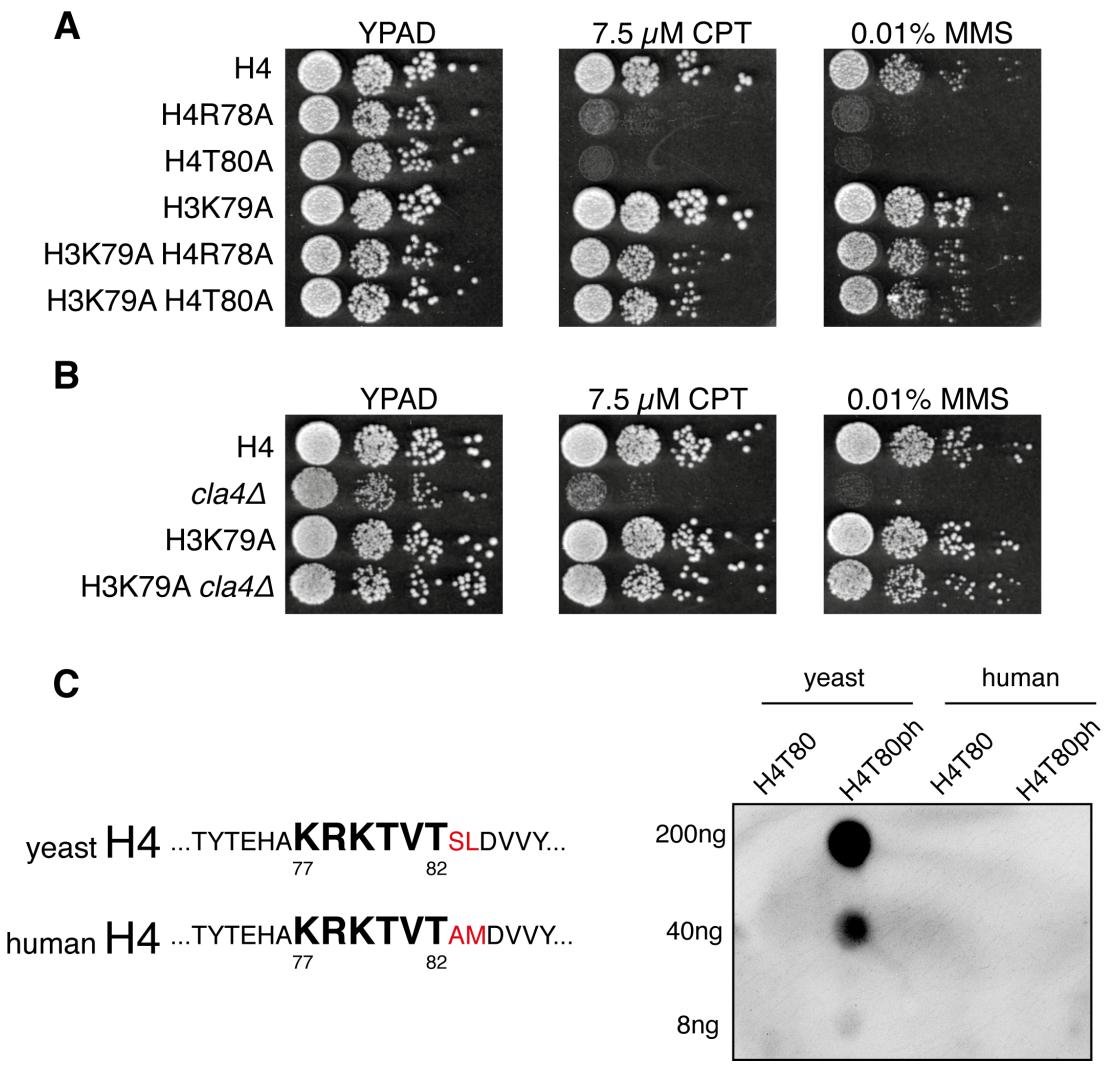

Figure S7. Mutation of H3K79A supresses H4T80A and *cla4Δ* mutant hypersensitivity to DNA damaging agents. Related to Figure 6. **(A)** and **(B)** Spot test for DNA damage sensitivity of different yeast mutants as indicated. 10-fold serial dilutions were used. **(C) Left** Comparison of yeast and human histone H4 L2 loop sequence. **Right** Dot-blot analysis using H4T80ph specific antibody. 5-fold serial dilutions of different peptides, as indicated, were spotted onto a PVDF membrane.

Strain	Genotype	Source
PTY1	*Mat a hht2,hhf2::natMX4*, *hht1,hhf1::kanMX4* pURA3-*HHT1-HHF1*	(Tessarz et al., 2014)
GMY041	PTY1 *cla4::hphMX4*	This study
GMY042	PTY1 *skm1::hphMX4*	This study
GMY043	PTY1 *ste20::hphMX4*	This study
GMY044	PTY1 *rad52::hphMX4*	This study
GMY045	PTY1 *RAD9-3HA::HIS3*	This study
GMY046	PTY1 *RTT107-3HA::HIS3*	This study
GMY047	PTY1 *dot1::HIS3*	This study
GMY048	PTY1 *cla4::hphMX4*, *dot1::HIS3*	This study
PTY2	*Mat a hht2,hhf2::KanMX4, hht1,hhf1::HIS3, hta1,htb1::NatMX6, hta2, htb2::hphMX6* pURA3-*HHT1-HHF1*	(Tessarz et al., 2014)
JKM179	*MATα ade1 leu2*-3,112, *lys5, trp1::hisG, ura3*-52, *hml::ADE1 hmr::ADE1 ade3::GAL::HO*	(Lee et al., 1998)
GMY049	JKM179 *CLA4-6HA::kanMX4*	This study
GMY050	JKM179 *cla4::hphMX4*	This study
GMY051	JKM179 *hht2,hhf2::natMX4*, *hht1,hhf1::kanMX4*	This study
GMY052	GMY051 *RTT107-6HA::hphMX4*	This study
GMY053	*Mat a hht2,hhf2::natMX4*, *hht1,hhf1::LEU2* pURA3-*HHT1-HHF1*	This study
GMY053	GMY053 *rad53-R605A::kanMX6*	
MKY5	*MATα ade2-1 can1-100 his3-11 leu2-3,112 trp1-1 ura3-1*	(Leung et al., 2016)
MKY1671	MKY5, *rtt107::kanMX6* *pRS315, RTT107-3XFLAG::natMX6*	(Leung et al., 2016)
MKY1672	MKY5, *rtt107::kanMX6* *pRS315, rtt107-K887M-3XFLAG::natMX6*	(Leung et al., 2016)
MKY1731	MKY5, *rtt107::KANMX6* *pRS315, rtt107-K426M-3XFLAG::NATMX6*	(Leung et al., 2016)

Table S1. Yeast Strains used in this study. Related to Key Resources Table. Strains are derivatives of W303. Histone point mutants were shuffled into yeast by counter-selection on 5-FOA.
